# A three-terminal magnetic thermal transistor

**DOI:** 10.1038/s41467-023-36056-4

**Published:** 2023-01-24

**Authors:** Lorenzo Castelli, Qing Zhu, Trevor J. Shimokusu, Geoff Wehmeyer

**Affiliations:** Mechanical Engineering, William Marsh Rice University, Houston, TX 77005 USA

**Keywords:** Mechanical engineering, Materials for devices

## Abstract

Three-terminal thermal analogies to electrical transistors have been proposed for use in thermal amplification, thermal switching, or thermal logic, but have not yet been demonstrated experimentally. Here, we design and fabricate a three-terminal magnetic thermal transistor in which the gate temperature controls the source-drain heat flow by toggling the source-drain thermal conductance from ON to OFF. The centimeter-scale thermal transistor uses gate-temperature dependent magnetic forces to actuate motion of a thermally conducting shuttle, providing thermal contact between source and drain in the ON state while breaking contact in the OFF state. We measure source-drain thermal switch ratios of 109 ± 44 in high vacuum with gate switching temperatures near 25 °C. Thermal measurements show that small heat flows into the gate can be used to drive larger heat flows from source to drain, and that the switching is reversible over >150 cycles. Proof-of-concept thermal circuit demonstrations show that magnetic thermal transistors can enable passive or active heat flow routing or can be combined to create Boolean thermal logic gates. This work will allow thermal researchers to explore the behavior of nonlinear thermal circuits using three-terminal transistors and will motivate further research developing thermal transistors for advanced thermal control.

## Abstract (web summary)

Thermal analogues to electrical transistors offer the potential for heat flow switching and amplification. Here, the authors demonstrate a macroscopic magnetic thermal transistor with applications in thermal control and thermal logic circuits.

## Introduction

Analogies between thermal and electrical components have proven to be useful in the development of switchable and nonlinear thermal devices^1–4^. In the typical mapping between thermal and electrical devices, temperature differences are analogous to voltage differences, and heat flows are analogous to electrical currents. For example, researchers have experimentally demonstrated two-terminal thermal diodes that display thermal rectification^5–10^, as well as two-terminal thermal switches that are used for thermal regulation^11–16^. Inspired by electrical device applications of diodes and switches, these thermal devices have been used in applications ranging from waste heat scavenging^17,18^ to solid-state cooling^19–21^ or battery thermal management^16,20,22^.

Despite the interest in thermal analogies to electrical devices, there are no experimentally demonstrated devices that are thermally analogous to an electrical transistor. Electrical field-effect transistors (FETs) are three-terminal transconductance devices in which the current flow between source and drain is controlled by the gate-drain or gate-source voltage^23^, while electrical bipolar junction transistors (BJTs) are three-terminal current amplification devices in which the collector current is controlled by the base-emitter current^24^. By analogy with these electrical devices, a thermal transistor is a three-terminal thermal element in which the temperature or heat flow at one terminal controls the heat flow between the other two terminals in a manner that leads to heat flow switching and amplification. Electrical transistors revolutionized society and have formed the backbone of modern computing and power transmission. Thermal researchers have proposed that thermal transistors could be used in precision thermal management^25^, advanced thermal sensing and integrated control^26^, and passive thermal logic/computation in harsh climates with no available electrical power^4,27,28^.

Previous researchers have computationally investigated three-terminal thermal transistors using mechanisms based on thermal radiation^25,26,29–34^, nonlinear phonon conduction in nanoscale systems^35–40^, nanoscale confined fluids^41,42^, quantum electronic systems^43–50^, and superconducting devices^51,52^. These proposed mechanisms all involve the concept of a negative differential thermal resistance (NDTR)^35,39^; using the terminology of a FET, NDTR refers to the regime in which increasing the gate temperature increases the heat flow from the source into the transistor at a fixed source temperature and drain temperature. Linear time-invariant thermal systems do not display NDTR, meaning that thermal nonlinearities are required to achieve thermal transistor action. As an aside, the term thermal transistor has also been used to describe experimental demonstrations of an electrically controlled two-terminal thermal switch^53–56^ or an electrical transistor with a highly temperature-dependent response^57^, though these devices are distinct from the three-thermal terminal transistor defined in this work. Despite this prior theoretical interest in three-terminal thermal transistor systems, no thermal transistor devices have been experimentally demonstrated, presumably due to the challenges in the fabrication and/or measurement of the previously proposed thermal transistor mechanisms.

Here, we fabricate a centimeter-scale three-terminal thermal transistor and use thermal measurements to establish the switching and amplification. Our magnetic thermal transistor uses the temperature (*T*) -dependent magnetization of a ferromagnetic material mounted on the gate terminal to control the steady-state position of a thermally bridging element connecting the thermal source to the thermal drain in the ON state. The transistor is a thermal transconductance device that exhibits NDTR when the gate temperature (*T*_g_) is larger than or comparable to the Curie temperature (${T}_{{{{{{\rm{Curie}}}}}}}$) of the ferromagnetic gate material, which in our case is a gadolinium foil with *T*_Curie_ = 23 °C. Our thermal measurements confirm the expected switching and amplification associated with transistor action. Like several other proposed thermal transistors using phase-change materials^25,26,32,34^, our magnetic transistor is binary in the sense that the source–drain thermal conductance can be toggled either ON or OFF via *T*_g_. We use reference bar thermal measurements to quantify the *T*_g_-controlled switching of the drain heat flow and achieve steady-state ON/OFF switching ratios of 109 ± 44 in high vacuum with small (7 °C) thermal hysteresis and durability over >150 gate-temperature thermal cycles. We investigate the behavior of the thermal transistor in several thermal circuits and show that the device enables active or passive heat flow routing for applications in controlled power generation and thermal storage. For more speculative applications in thermal computing or thermal sensing, the transistors can be combined in series to create a thermal AND logic gate or combined in parallel to create a thermal OR logic gate. Overall, this magnetic thermal transistor demonstration provides an experimental platform to explore three-terminal thermal switching and amplification of heat flows and motivates further research implementing thermal transistors for improved control of engineering systems.

## Results

### Thermal transistor concept

**Fig. 1:**
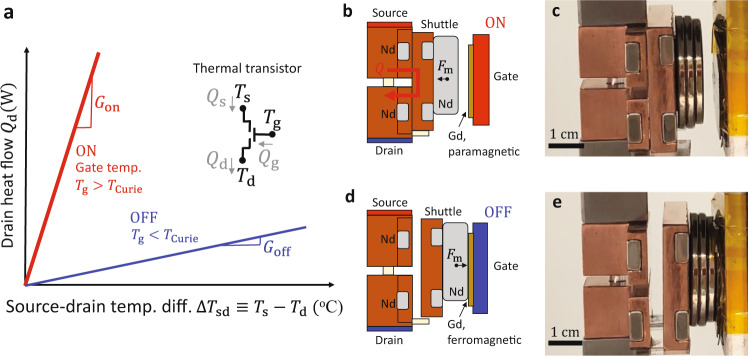
Thermal transistor concept and implementation. **a** In our three-terminal thermal transistor, the drain heat flow ${Q}_{{{{{{\rm{d}}}}}}}$ is controlled by the gate temperature ${T}_{{{{{{\rm{g}}}}}}}$ at a fixed source-drain temperature difference $\Delta {T}_{{{{{{\rm{sd}}}}}}}$. The mechanism leverages ${T}_{{{{{{\rm{g}}}}}}}$-dependent magnetic forces between gadolinium (Gd) and neodymium (Nd) magnets to achieve source–drain thermal conductances $G\equiv {Q}_{{{{{{\rm{d}}}}}}}/\Delta {T}_{{{{{{\rm{sd}}}}}}}$ that are larger in the high-${T}_{{{{{{\rm{g}}}}}}}$ ON state (${G}_{{{{{{\rm{on}}}}}}},$red) than in the low-${T}_{{{{{{\rm{g}}}}}}}$ OFF state (${G}_{{{{{{\rm{off}}}}}}},$blue). **b** Schematic and **c** optical image of the transistor in the high-${T}_{{{{{{\rm{g}}}}}}}$ON state. Attractive forces between permanent Nd magnets pull a copper shuttle into thermal contact with the source and drain, leading to a large ${G}_{{{{{{\rm{on}}}}}}}$ enabled by conduction through the shuttle. Because ${T}_{{{{{{\rm{g}}}}}}}$ is larger than the Gd Curie temperature ${T}_{{{{{{\rm{Curie}}}}}}}={23}\,^{{{{{{\rm{o}}}}}}}{{{{{\rm{C}}}}}}$, the paramagnetic Gd on the gate experiences a weak magnetic interaction with the shuttle. **d** Schematic and **e** image of the transistor in the OFF state in which ${T}_{{{{{{\rm{g}}}}}}}\, < \,{T}_{{{{{{\rm{Curie}}}}}}}$. Strong attractive magnetic forces between the ferromagnetic Gd and shuttle Nd magnets pull the shuttle out of contact with the source and drain, leading to a low ${G}_{{{{{{\rm{off}}}}}}}$ due to parasitic conduction and radiation from the source to drain.

Figure 1 introduces the concept and design of the magnetic thermal transistor, which has a steady-state thermal transfer function illustrated in Fig. 1a. The thermal transistor schematic inset in Fig. 1a labels the key source, drain, and gate temperatures $({T}_{{{{{{\rm{s}}}}}}},{T}_{{{{{{\rm{d}}}}}}},{T}_{{{{{{\rm{g}}}}}}})$ and heat flows $({Q}_{{{{{{\rm{s}}}}}}},{Q}_{{{{{{\rm{d}}}}}}},{Q}_{{{{{{\rm{g}}}}}}})$. The effects of thermal losses from the device to the ambient are important for quantitative measurements and are considered below, but do not modify the qualitative transistor performance presented here. The unique aspect of the transistor is the ability to use the gate temperature *T*_g_ to switch the drain heat flow ${Q}_{{{{{{\rm{d}}}}}}}$at a fixed source–drain temperature difference $\Delta {T}_{{{{{{\rm{sd}}}}}}}\equiv$ $({T}_{{{{{{\rm{s}}}}}}}-{T}_{{{{{{\rm{d}}}}}}})$. Here, we focus on the case where $\Delta {T}_{{{{{{\rm{sd}}}}}}}\, > \,0$ for clarity because the device functions similarly in the reverse-biased case where $\Delta {T}_{{{{{{\rm{sd}}}}}}}\, < \,0$. In an idealized magnetic transistor, the thermal conductance ${G\equiv Q}_{{{{{{\rm{d}}}}}}}/\Delta {T}_{{{{{{\rm{sd}}}}}}}$ (graphically, the slope of the lines in Fig. 1a) can be toggled ON by setting ${T}_{{{{{{\rm{g}}}}}}}\, > \,{T}_{{{{{{\rm{Curie}}}}}}}$ (red) and OFF by setting ${T}_{{{{{{\rm{g}}}}}}}\, < \,{T}_{{{{{{\rm{Curie}}}}}}}$ (blue). As discussed below, the ON–OFF and OFF–ON transition temperatures ${T}_{{{{{{\rm{on}}}}}}-{{{{{\rm{off}}}}}}}$ and ${T}_{{{{{{\rm{off}}}}}}-{{{{{\rm{on}}}}}}}$ are not identical to ${T}_{{{{{{\rm{Curie}}}}}}}$ and can be tuned via the transistor geometry; the simplified representation in Fig. 1a is intended to illustrate the fundamental magnetic origin of the thermal switching mechanism. The thermal conductance switching ratio $\gamma \equiv {G}_{{{{{{\rm{on}}}}}}}/{G}_{{{{{{\rm{off}}}}}}}$ is one primary metric of transistor performance. The thermal transfer function in Fig. 1a has several similarities to a common representation of the electrical transfer function of a field-effect transistor (FET), in which the source-drain electrical current is plotted as a function of the source–drain voltage for a range of gate voltages to illustrate the switching capabilities. Two important differences between the magnetic thermal transistor and the electric FET transfer functions are: (1) The thermal transistor displays only a binary switching instead of a continuous tuning with gate setting, and (2) the thermal transistor does not exhibit saturation at large source–drain bias. In addition, in the electrical transistor, the gate-source or gate-drain voltage difference determines the gating; in the thermal transistor, the absolute magnitude of the gate temperature itself determines the ON or OFF performance, rather than the gate-drain or gate-source temperature differences.

Figure 1b and c show a schematic and an image, respectively, of the magnetic thermal transistor biased in the ON state. Supplementary Movie 1 also shows the transistor switching between the ON and OFF states as a function of *T*_g_. The device consists of stationary thermally conducting elements as the thermal source and drain, a stationary gadolinium (Gd) foil mounted on the gate terminal, and a mobile element that we refer to as a shuttle. The steady-state position of the shuttle is controlled by *T*_g_. In the high-*T*_g_ ON state shown in Fig. 1b and c, the Gd foil is in a paramagnetic state because ${T}_{{{{{{\rm{g}}}}}}}\, > \,{T}_{{{{{{\rm{Curie}}}}}}}$, and the permanent neodymium (Nd) alloy magnets mounted on the source, drain, and shuttle do not interact magnetically with the paramagnetic Gd foil. These Nd magnets are oriented such that the shuttle is magnetically attracted to the source and drain contacts, as indicated in Fig. 1b by the direction of the magnetic force ${F}_{{{{{{\rm{m}}}}}}}$. In this ON state, the shuttle provides a low-thermal resistance pathway to heat conduction from source to drain. The thermal modeling discussed in Supplementary Note 1 indicates that the limiting thermal resistance in the ON state is due to the thermal contact resistances at the shuttle–source and shuttle–drain interfaces. These contact resistances arise from the imperfect contact between mated engineering surfaces and are sensitive to the contact pressures, surface polish, ambient pressure (i.e. presence or absence of air pockets trapped between surface asperities), and selection of thermal interface materials^58^. In contrast, the gate is thermally isolated from the source, drain, or shuttle in the ON state, as heat is only transferred via radiation or through the air between the gate and shuttle. This thermal isolation is desirable to decrease the ON-state gate heat flow ${Q}_{{{{{{\rm{g}}}}}}}$ illustrated in Fig. 1a inset.

Figure 1d and e show a schematic and an image, respectively, of the transistor in the low-*T*_g_ OFF state. Because ${T}_{{{{{{\rm{g}}}}}}}\, < \,{T}_{{{{{{\rm{Curie}}}}}}}$, the Gd foil is in a ferromagnetic state and interacts magnetically with the fields generated by the Nd magnets. The magnetization of the Nd magnets depends weakly on *T* in these measurements, while the strong *T*-dependent magnetic response of the Gd foil provides the key thermomagnetic actuation of the transistor^10^. At sufficiently low *T*_g_, the magnetic permeability of the Gd foil is large enough to pull the shuttle into a steady-state contact with the gate, as indicated by the different shuttle location and direction of ${F}_{{{{{{\rm{m}}}}}}}$ in the OFF state of Fig. 1d as compared to the ON state of Fig. 1b. The critical gate temperature required to actuate switching depends on the ON-state gap size between gate and shuttle, the positioning and strength of the Nd magnets, and the thickness of the Gd foil. Unlike previous thermomagnetic demonstrations in which the Gd is mounted on the moving element^5,10^, the transistor shuttle does not oscillate but instead remains connected to the gate at all times at low *T*_g_. Because the shuttle is no longer providing thermal contact between the source and drain in the OFF state, heat flows from source to drain only parasitically via conduction through the mechanical supports, conduction/convection through the air, and radiation. Thermal modeling in Supplementary Note 1 shows that parasitic radiation and parasitic conduction through mechanical support are the dominant heat transfer mechanisms in a vacuum, whereas parasitic conduction through the air is the dominant mechanism in air measurements. The gate terminal remains thermally isolated from the source and drain in the OFF state, as the heat transfer pathways from the gate to the source and drain include only radiation and conduction/convection through the air.

### Thermal measurements

**Fig. 2:**
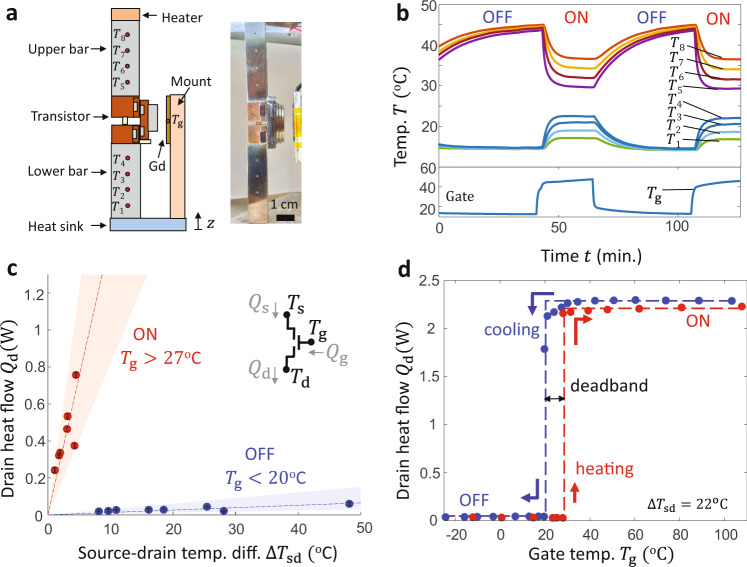
Thermal transistor measurements. **a** Schematic (left) and optical image (right) of reference bar measurements used to quantify the thermal transistor performance under high vacuum. **b** Time-dependent measurements show transistor cycling over two OFF to ON cycles. We set the transistor state by controlling ${T}_{{{{{{\rm{g}}}}}}}$ (bottom panel) and measure the reference bar temperatures ${T}_{1}-{T}_{8}$ (top panel) to extract ${Q}_{{{{{{\rm{d}}}}}}},$ ${T}_{{{{{{\rm{s}}}}}}}$, and ${T}_{{{{{{\rm{d}}}}}}}$. **c** Reference bar measurements show that the transistor achieves a ${T}_{{{{{{\rm{g}}}}}}}$-controlled thermal switching ratio ${G}_{{{{{{\rm{on}}}}}}}/{G}_{{{{{{\rm{off}}}}}}}=109\pm 44$, demonstrating the excellent source–drain switching via the gate terminal setting. Error bars represent standard deviations of ${Q}_{{{{{{\rm{d}}}}}}}$ arising from the thermocouple measurements. Lines are linear fits used to extract the thermal conductance, and shading represents the trial-to-trial standard deviation of ${G}_{{{{{{\rm{on}}}}}}}$ and ${G}_{{{{{{\rm{off}}}}}}}$. **d** Steady-state ${Q}_{{{{{{\rm{d}}}}}}}$ measurements at fixed $\Delta {T}_{{{{{{\rm{sd}}}}}}}=22\pm {4}\,^{{{{{{\rm{o}}}}}}}{{{{{\rm{C}}}}}}$ show a transition temperature ${T}_{{{{{{\rm{on}}}}}}-{{{{{\rm{off}}}}}}}=2{0}\,^{{{{{{\rm{o}}}}}}}{{{{{\rm{C}}}}}}$ on cooling (blue) and ${T}_{{{{{{\rm{off}}}}}}-{{{{{\rm{on}}}}}}}={27}\,^{{{{{{\rm{o}}}}}}}{{{{{\rm{C}}}}}}$ on heating (red), leading to a narrow thermal deadband of ${7}\,^{{{{{{\rm{o}}}}}}}{{{{{\rm{C}}}}}}$. Lines are guide to eye.

Figure 2 shows the measurements of the thermal transistor transfer function obtained using the reference bar method in Fig. 2a. The measurement is performed under a high vacuum to eliminate convection losses to the surrounding air. Figure 2b shows typical measured thermal data obtained during two OFF–ON cycles of our reference bar experiment. We control *T*_g_ (bottom panel of Fig. 2b) to switch the transistor ON and OFF, measure the temperatures ${T}_{1}-{T}_{8}$ (top panel of Fig. 2b) in the reference bars as a function of time, and use the steady-state reference bar temperatures to extract $\Delta {T}_{{{{{{\rm{sd}}}}}}}$ and ${Q}_{{{{{{\rm{d}}}}}}}$. In the ON state, the large thermal conductance leads to a large heat flow through the reference bars (manifested in the experiment by sharp spatial temperature gradients within each reference bar) and a relatively small temperature drop across the transistor (as seen by the relatively small $({T}_{5}-{T}_{4})$ in the ON state of Fig. 2b). In the OFF state, the small thermal conductance of the transistor leads to a smaller heat flow (as seen by the smaller differences within the top reference temperatures ${T}_{1}-{T}_{4}$and within the bottom reference bar temperatures ${T}_{5}-{T}_{8}$) and a larger temperature drop across the transistor, demonstrating the strong gate-driven transistor switching.

Figure 2c shows the reference bar measurements of the thermal transistor transfer function, providing quantitative backing to the qualitative transfer function introduced schematically in Fig. 1a. The data points in Fig. 2c represent steady-state measurements of ${Q}_{{{{{{\rm{d}}}}}}}$ and $\Delta {T}_{{{{{{\rm{sd}}}}}}}$at different *T*_g_, and error bars represent the uncertainties in ${Q}_{{{{{{\rm{d}}}}}}}$ extracted from the temperature measurements, as discussed in Supplementary Note 2. ${T}_{{{{{{\rm{s}}}}}}}$ and ${T}_{{{{{{\rm{d}}}}}}}$ were found by linearly extrapolating the steady-state temperature profile on the top and bottom reference bar to the boundaries of the device; ${T}_{{{{{{\rm{s}}}}}}}$ is the extrapolated temperature at the top reference-bar/transistor interface, and ${T}_{{{{{{\rm{d}}}}}}}$ is the extrapolated temperature at the bottom reference-bar/transistor interface. The abscissa of Fig. 2c represents the source–drain temperature difference ${\triangle T}_{{{{{{\rm{sd}}}}}}}=({T}_{{{{{{\rm{s}}}}}}}-{T}_{{{{{{\rm{d}}}}}}})$. The specific values of ${T}_{{{{{{\rm{s}}}}}}}$ and ${T}_{{{{{{\rm{d}}}}}}}$ vary between different experimental measurements and are included in Supplementary Tables 1 and 2 along with the extracted values of $\Delta {T}_{{{{{{\rm{sd}}}}}}}$ and *Q*. The ON state measurements (red points and line) are performed with *T*_g_ larger than the transition temperature ${T}_{{{{{{\rm{off}}}}}}-{{{{{\rm{on}}}}}}}$= 27 °C and display a thermal conductance of ${G}_{{{{{{\rm{on}}}}}}}=0.15\pm 0.04\frac{{{{{{\rm{W}}}}}}}{{{{{{\rm{K}}}}}}}$, leading to large drain heat flows up to ${Q}_{{{{{{\rm{d}}}}}}}=0.76\,{{{{{\rm{W}}}}}}$ at moderate Δ*T*_sd_ = 4.5 °C. We combined these ${G}_{{{{{{\rm{on}}}}}}}$ measurements with finite element method calculations to find the thermal contact resistance for a unit area at the shuttle–source and shuttle–drain contact is ${R}_{{{{{{\rm{contact}}}}}}}\cong 4*{10}^{-3}\,{{{{{\rm{m}}}}}}^{2}\,{{{{{\rm{K}}}}}}/{{{{{\rm{W}}}}}}$. As discussed in Supplementary Note 1, this contact resistance value is of an expected order-of-magnitude based on the predictions of thermal models describing conduction between contacting surface asperities^59^.

In contrast to the relatively large ON state conductance, the OFF state measurements (red points and line in Fig. 2c) performed with *T*_g_ smaller than the transition temperature ${T}_{{{{{{\rm{on}}}}}}-{{{{{\rm{off}}}}}}}=$ 20 °C have an ultralow thermal conductance of ${G}_{{{{{{\rm{off}}}}}}}=0.0013\pm 0.0004\,{{{{{\rm{W}}}}}}/{{{{{\rm{K}}}}}}$, leading to much lower ${Q}_{{{{{{\rm{d}}}}}}}=0.06\,{{{{{\rm{W}}}}}}$ even at a large $\Delta {T}_{{{{{{\rm{sd}}}}}}}=$ 48 °C. In Supplementary Note 1 we calculate that conduction through the mechanical support dominates the parasitic source–drain heat transfer in vacuum, although parasitic radiation becomes a significant contribution at higher temperatures (up to 43% of total source-drain heat transfer for the largest ${T}_{\rm {{s}}}$ values considered here). The switch ratio of the device is$\,\gamma=\frac{{G}_{{{{{{\rm{on}}}}}}}}{{G}_{{{{{{\rm{off}}}}}}}}=109\pm 44$; the uncertainty in *γ* results from a roughly equal contribution between the uncertainties in ${G}_{{{{{{\rm{off}}}}}}}$ and ${G}_{{{{{{\rm{on}}}}}}}$, as discussed in Supplementary Note 2. The trial-to-trial standard deviation in the ${G}_{{{{{{\rm{off}}}}}}}$ and ${G}_{{{{{{\rm{on}}}}}}}$ values are indicated in Fig. 2c by the blue and red shading, respectively. The uncertainty in ${G}_{{{{{{\rm{off}}}}}}}$arises due to the errors involved in measuring small ${Q}_{{{{{{\rm{d}}}}}}}$ in the reference bar method, as indicated by the error bars on each ${Q}_{{{{{{\rm{d}}}}}}}$ measurement in Fig. 2c (black lines, typically comparable to the size of the data point). In contrast, the uncertainty in ${G}_{{{{{{\rm{on}}}}}}}$ is not due to uncertainty in measuring ${Q}_{{{{{{\rm{d}}}}}}}$ (e.g., in Fig. 2c the error bars in the ON state measurements are much smaller than the magnitude of ${Q}_{{{{{{\rm{d}}}}}}}$), but rather to deviations in thermal contact conductance between each trial. As the shuttle makes and breaks contact with the source/drain, different cycles will have slight modifications in the surface contact pressures that lead to different trial-to-trial ${G}_{{{{{{\rm{on}}}}}}}$. In the OFF state, we expect that ${G}_{{{{{{\rm{off}}}}}}}$ does not physically vary from trial-to-trial, since the OFF state conductance is not sensitive to contact pressures between mating surfaces. Our *γ* for the thermal transistor is orders of magnitude smaller than the electrical current switching ratio observed in FETs^60^, because parasitic thermal conduction and radiation are difficult to eliminate and the pressed mechanical contacts have limited contact conductance in the ON state. Supplementary Fig. 6 compares our transistor switching ratios to those measured in existing two-terminal heat switches and shows that several conduction-based thermal switches have *γ* > 100^61^ while many other demonstrations have much smaller *γ*^62,63^.

We primarily focus on the measured conductances ${G}_{{{{{{\rm{on}}}}}}}$ and ${G}_{{{{{{\rm{off}}}}}}}$ because these quantities are useful in the modeling of the thermal transistor circuits shown in the section “Proof-of-concept transistor circuits” below. Alternate metrics for assessing steady-state heat transfer can also be useful in comparing the transistor to other thermal devices; for example, normalizing the thermal conductance by the reference bar cross-sectional area ${A}_{{{{{{\rm{rb}}}}}}}$ leads to effective heat transfer coefficients of $\frac{{G}_{{{{{{\rm{on}}}}}}}}{{A}_{{{{{{\rm{rb}}}}}}}}=930\,{{{{{\rm{W}}}}}}/{{{{{\rm{m}}}}}}^{2}{{{{{\rm{K}}}}}}$ and $\frac{{G}_{{{{{{\rm{off}}}}}}}}{{A}_{{{{{{\rm{rb}}}}}}}}=8\,{{{{{\rm{W}}}}}}/{{{{{\rm{m}}}}}}^{2}{{{{{\rm{K}}}}}}$. Similarly, the effective ON-state thermal conductivity $\frac{{G}_{{{{{{\rm{on}}}}}}}{L}_{{{{{{\rm{t}}}}}}}}{{A}_{{{{{{\rm{rb}}}}}}}}$ of the transistor is $23\,{{{{{\rm{W}}}}}}/{{{{{\rm{m}}}}}}\,{{{{{\rm{K}}}}}}$ and the effective OFF-state thermal conductivity is $\frac{{G}_{{{{{{\rm{off}}}}}}}{L}_{{{{{{\rm{t}}}}}}}}{{A}_{{{{{{\rm{rb}}}}}}}}=0.2{{{{{\rm{W}}}}}}/{{{{{\rm{m}}}}}}\,{{{{{\rm{K}}}}}}$, where ${L}_{{{{{{\rm{t}}}}}}}$ is the total length of the transistor spanning the source to drain. These alternate approaches for assessing the ON and OFF state conductances emphasize that the transistor is an excellent thermal insulator in the OFF state (with an effective thermal conductivity similar to that of common polymers), and a modest thermal conductor in the ON state (with an effective thermal conductivity similar to that of steel). This calculation also emphasizes that even though the transistor is made of high thermal conductivity copper, the effects of the contact resistances at the source–shuttle and drain–shuttle interfaces reduce the effective conductivity well below the intrinsic thermal conductivity of copper (~400 $\frac{{{{{{\rm{W}}}}}}}{{{{{{\rm{m}}}}}}.{{{{{\rm{K}}}}}}}$), as expected for pressed solid–solid contacts in a vacuum environment.

Figure 2d shows our steady-state reference bar measurements to quantify the switching temperatures (${T}_{{{{{{\rm{on}}}}}}-{{{{{\rm{off}}}}}}}$ and ${T}_{{{{{{\rm{off}}}}}}-{{{{{\rm{on}}}}}}}$) and the transistor NDTR. We measure ${Q}_{{{{{{\rm{d}}}}}}}$ as a function of *T*_g_ ranging from −24 °C to +103 °C at fixed $\Delta {T}_{{{{{{\rm{sd}}}}}}}=$22 ± 4 °C. In the cooling measurements (blue points, line as guide to eye), the transistor is initially prepared in the ON state with $T_{{{{{\rm{g}}}}}}$ = 103 °C and data is acquired for decreasing *T*_g_. Figure 2d shows that this steady-state ${Q}_{{{{{{\rm{d}}}}}}}$ obtained during the cooling cycle is constant near 2.3 W for all *T*_g_ > 30 °C, demonstrating the thermal isolation between the gate and drain. ${Q}_{{{{{{\rm{d}}}}}}}$ decreases slightly from 2.3 to 2.1 W as *T*_g_ decreases from 30 to 20 °C, which we attribute to the enhanced magnetization of the Gd foil at lower *T*_g_, which acts to reduce the contact pressure and thermal contact conductance between the shuttle and source/drain. When *T*_g_ < 20 °C, the transistor fully switches to the OFF state and ${Q}_{{{{{{\rm{d}}}}}}}\cong 0.03\,{{{{{\rm{W}}}}}}$ for all *T*_g_ upon further cooling to −24 °C. We measure a very similar OFF state ${Q}_{{{{{{\rm{d}}}}}}}$ during the subsequent heating cycle (red points and line) until the transistor switches ON at a temperature of *T*_g_ = 27 °C. In the experiment of Fig. 2d, we maintain ${\Delta T}_{{{{{{\rm{sd}}}}}}}$ within 4 °C of the average setpoint of 22 °C by iteratively changing the input power to the source heater and monitoring the steady-state cut-bar temperatures. Large differences in the source input power to maintain a fixed $\Delta {T}_{{{{{{\rm{sd}}}}}}}$are needed in the ON and OFF state, because *G* varies dramatically between these states.

The thermal deadband $\left({T}_{{{{{{\rm{off}}}}}}-{{{{{\rm{on}}}}}}}-{T}_{{{{{{\rm{on}}}}}}-{{{{{\rm{off}}}}}}}\right)={7}\deg {{{{{\rm{C}}}}}}$ in this demonstration is similar to the degree of hysteresis observed in several existing two-terminal thermal switches^64,65^. We attribute the hysteresis to the effects of stiction and to shuttle-location dependent magnetic forces. At fixed *T*_g_, the Gd-shuttle attractive magnetic force ${F}_{{{{{{\rm{Gd}}}}}}-{{{{{\rm{Nd}}}}}}}$is smaller in magnitude when the shuttle is contacting the source-drain (ON state position) than when the shuttle is contacting the gate (OFF state position) because the shuttle is closer to the source-drain Nd magnets and farther from the gate Gd foil in the ON state than in the OFF state. Lower *T*_g_ are therefore needed to obtain a desired ${F}_{{{{{{\rm{Gd}}}}}}-{{{{{\rm{Nd}}}}}}}$ and actuate switching from ON–OFF at large Gd-shuttle gap sizes *d*. Supplementary Fig. 1 shows our measurements confirming that the deadband increases with increasing *d*; for example, increasing *d* from 0.7 to 3.8 mm increases ${T}_{{{{{{\rm{off}}}}}}-{{{{{\rm{on}}}}}}}$ from 31.4 to 47.5 °C and decreases ${T}_{{{{{{\rm{on}}}}}}-{{{{{\rm{off}}}}}}}$from 25.4 to 14.5 °C. The gap size in the experiment of Fig. 2d was smaller than 0.7 mm. In the small *d* limit (not considered experimentally here), the deadband could plausibly also be influenced by the intrinsic hysteresis of the ferromagnetic-paramagnetic Gd phase transition, or by stiction forces that act to resist actuation. Lastly, Fig. 2d shows that the ON-state ${Q}_{{{{{{\rm{d}}}}}}}$ is independent of *T*_g_ upon heating to 103 °C. The ON-state ${Q}_{{{{{{\rm{d}}}}}}}$ is 4% smaller in the heating cycle (red) than in the cooling cycle (blue). We attribute this minor difference in the drain heat flows to slightly different shuttle contact configurations after thermal cycling, which would result in small differences in ${R}_{{{{{{\rm{contact}}}}}}}$ and ${Q}_{{{{{{\rm{d}}}}}}}$ between thermal cycles. Our primary focus is on the conductance measurements outside of the thermal deadband. At temperatures within or near the thermal deadband, the gadolinium foil is transitioning from a high-*T* paramagnetic to low-*T* ferromagnetic state. The magnetization changes continuously with *T* during this second-order phase transition, which modifies the ON state contact pressures and contact conductance in a manner that reduces *Q* in Fig. 2d. Note that these measurements are in steady-state, meaning that thermal transients associated with magnetocaloric effects in the Gd^66^ do not influence our transistor conductance measurements. Supplementary Fig. 2 shows the *T*_g_-dependent thermal conductance values obtained from the measurements in Fig. 2d, and confirms that ${G}_{{{{{{\rm{on}}}}}}}$and ${G}_{{{{{{\rm{off}}}}}}}$ do not depend strongly on *T*_g_ outside of the thermal deadband region. Our reference bar apparatus is optimized for steady-state measurements, meaning that we are unable to accurately extract any time-varying thermal conductance of the device from the reference bar measurements (due, for example, to thermal diffusion timescales within the device). Our transient measurements in the air discussed below provide more information on the thermal time constants of the transistor.

Figure 2d also demonstrates the magnetic thermal transistor’s negative differential thermal resistance. When the gate temperature increases above 27 °C during the heating cycle (red), the source–drain thermal resistance dramatically decreases with increasing *T*_g_, as manifested by the large increase in ${Q}_{{{{{{\rm{d}}}}}}}$ for ${T}_{{{{{{\rm{g}}}}}}}\, > \,{T}_{{{{{{\rm{off}}}}}}-{{{{{\rm{on}}}}}}}$ upon heating. Mathematically, the differential thermal resistance of the source ${R{{\hbox{'}}}}\equiv -{(\frac{{{{{{\rm{d}}}}}}{Q}_{{{{{{\rm{s}}}}}}}}{{{{{{\rm{d}}}}}}{T}_{{{{{{\rm{g}}}}}}}})}^{-1}|_{{T}_{{{{{{\rm{s}}}}}}},{T}_{{{{{{\rm{d}}}}}}}}$ is negative-valued as *T*_g_ crosses ${T}_{{{{{{\rm{off}}}}}}-{{{{{\rm{on}}}}}}}$; here, the prime notation distinguishes the differential thermal resistance from the total thermal resistance $R=1/G$ considered in the thermal circuits below. In linear thermal systems such as the three-terminal connection of linear thermal resistors considered in Supplementary Note 4 and Supplementary Fig. 3, *R*′ is positive-valued, which further implies that the thermal amplification ratio $\beta \equiv \Delta {Q}_{{{{{{\rm{d}}}}}}}/\Delta {Q}_{{{{{{\rm{g}}}}}}}$ is always smaller than unity in linear thermal systems. Here, $\Delta {Q}_{{{{{{\rm{d}}}}}}}$ is the change in the drain heat flow induced by $\Delta {Q}_{{{{{{\rm{g}}}}}}}$, the change in the gate heat flow. In contrast, the NDTR observed in our scenario in Fig. 2d indicates that the magnetic transistor can indeed amplify heat currents, meaning that a small increase in the heat flow into the gate can drive a larger heat flow from source to drain.

**Fig. 3:**
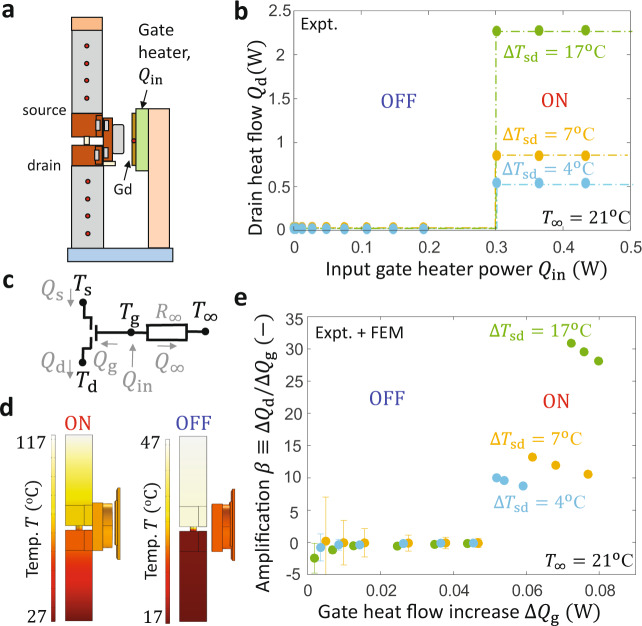
Transistor heat flow amplification. **a** The transistor can use a small gate heat flow ${Q}_{{{{{{\rm{g}}}}}}}$to drive a large source-drain heat flow. To quantify this thermal amplification, we mount the Gd on a heater and use the reference bar method in vacuum to measure ${Q}_{{{{{{\rm{d}}}}}}}$ as a function of the gate input power ${Q}_{{{{{{\rm{in}}}}}}}$. **b** A gate input power of ${Q}_{{{{{{\rm{in}}}}}}}=0.3\,{{{{{\rm{W}}}}}}$ heats the gate above ${T}_{{{{{{\rm{off}}}}}}-{{{{{\rm{on}}}}}}}$ and drives drain heat flows as large as ${Q}_{{{{{{\rm{d}}}}}}}=2.2$ W at $\Delta {T}_{{{{{{\rm{sd}}}}}}}={17}{\deg{{{{\rm{C}}}}}}$. At ${Q}_{{{{{{\rm{in}}}}}}}\, < \,0.2\,{{{{{\rm{W}}}}}}$ and ${T}_{{{{{{\rm{g}}}}}}}\, < \,{T}_{{{{{{\rm{off}}}}}}-{{{{{\rm{on}}}}}}}$ the transistor is OFF for all $\Delta {T}_{{{{{{\rm{sd}}}}}}}$. Lines are guides to the eye. **c** The true gate heat flow ${Q}_{{{{{{\rm{g}}}}}}}$ is smaller than ${Q}_{{{{{{\rm{in}}}}}}}$ due to thermal losses to the surroundings ${Q}_{\infty }$, as illustrated in a thermal circuit representation. **d** We use finite element method (FEM) calculations to estimate ${Q}_{{{{{{\rm{g}}}}}}}$ and ${Q}_{\infty }$ in the measurements, as shown here for ${T}_{\infty }={21}{\deg{{{{\rm{C}}}}}}$, ON-state ${T}_{{{{{{\rm{g}}}}}}}={67}{\deg{{{{\rm{C}}}}}}$ (left), and OFF-state ${T}_{{{{{{\rm{g}}}}}}}={25}{\deg{{{{\rm{C}}}}}}$ (right). (e) We combine these FEM results from **d** with our measurements from **b** to find that the intrinsic thermal amplification $\beta=\Delta {Q}_{{{{{{\rm{d}}}}}}}/{\Delta Q}_{{{{{{\rm{g}}}}}}}$ ranges from $8$ to $31$ for $\Delta {Q}_{{{{{{\rm{g}}}}}}}\, > \,0.05\,{{{{{\rm{W}}}}}}$. As expected, $\beta$ approaches zero for smaller $\Delta {Q}_{{{{{{\rm{g}}}}}}}$ values when the transistor is in the OFF state and ${T}_{{{{{{\rm{g}}}}}}}\, < \,{T}_{{{{{{\rm{off}}}}}}-{{{{{\rm{on}}}}}}}$. Error bars represent uncertainty in $\Delta {Q}_{{{{{{\rm{d}}}}}}}$ due to thermocouple measurements.

Figure 3 quantifies the steady-state heat flow amplification of the thermal transistor. This amplification depends strongly on the heat flow between gate and source/drain terminals ${Q}_{{{{{{\rm{g}}}}}}}$, as well as the interaction between the gate and surroundings at a temperature ${T}_{\infty }$. Figure 3a illustrates our reference bar measurements of the drain heat flow ${Q}_{{{{{{\rm{d}}}}}}}$ as a function of the input electrical power to a gate heater ${Q}_{{{{{{\rm{in}}}}}}}$ for different values of the source–drain temperature difference $\Delta {T}_{{{{{{\rm{sd}}}}}}}$. Figure 3b shows that increasing the steady-state ${Q}_{{{{{{\rm{in}}}}}}}$ from 0 to 0.3 W $(\Delta {Q}_{{{{{{\rm{in}}}}}}}=0.3\,{{{{{\rm{W}}}}}})$ acts to heat up the gate above ${T}_{{{{{{\rm{off}}}}}}-{{{{{\rm{on}}}}}}}$ and to switch the transistor ON. This gate-induced switching drives a larger increase in ${Q}_{{{{{{\rm{d}}}}}}}$ such that $\Delta {Q}_{{{{{{\rm{d}}}}}}}$ can be larger than $\Delta {Q}_{{{{{{\rm{in}}}}}}}$, which is not possible in linear thermal systems. For example, at $\Delta {T}_{{{{{{\rm{sd}}}}}}}={17}\deg {{{{{\rm{C}}}}}}$, $\Delta {Q}_{{{{{{\rm{d}}}}}}}$ is larger than $2.2\,{{{{{\rm{W}}}}}}$, leading to device-level effective amplification ratios of $\Delta {Q}_{{{{{{\rm{d}}}}}}}/\Delta {Q}_{{{{{{\rm{in}}}}}}}=7.5$. The true amplification ratio of the device is even larger than 7.5, because ${Q}_{{{{{{\rm{in}}}}}}}$ is larger than ${Q}_{{{{{{\rm{g}}}}}}}$ due to thermal losses from the heater to the surroundings at a temperature ${T}_{\infty }={21}\deg {{{{{\rm{C}}}}}}$. The effects of the exchange with the surroundings are represented in the thermal circuit of Fig. 3c by the gate-to-surroundings thermal resistance ${R}_{\infty }$. We estimate the intrinsic gate heat flow ${Q}_{{{{{{\rm{g}}}}}}}$ and the heat loss ${Q}_{\infty }$ in the ON and OFF state using finite-element method (FEM) calculations of the steady-state temperature profiles shown in Fig. 3d. These FEM calculations include the radiative exchanges between components and surroundings as well as the conduction through the transistor. Considering a representative OFF-state measurement in which ${T}_{{{{{{\rm{g}}}}}}}={50}\deg {{{{{\rm{C}}}}}}$, ${T}_{8}={47}\deg {{{{{\rm{C}}}}}}$, and ${T}_{1}={11}\deg {{{{{\rm{C}}}}}}$, our FEM simulations show that 25% of the heat is transferred to the transistor body via radiation $({Q}_{{{{{{\rm{g}}}}}}})$, whereas the remainder of the input heat flow is lost to the surroundings.

We combine the experimental ${Q}_{{{{{{\rm{d}}}}}}}$ measurements of Fig. 3b with the FEM modeling of ${Q}_{{{{{{\rm{g}}}}}}}$ from Fig. 3d to extract the intrinsic thermal amplification ratio $\beta=\Delta {Q}_{{{{{{\rm{d}}}}}}}/\Delta {Q}_{{{{{{\rm{g}}}}}}}$ in Fig. 3e. Here, $\Delta {Q}_{{{{{{\rm{d}}}}}}}$ and $\Delta {Q}_{{{{{{\rm{g}}}}}}}$ are the increase in the drain and gate heat flows compared to the base values in which no input power is applied to the gate. We designed the transistor to eliminate the conduction pathway through the gate, but the remaining radiation component of the gate heat flow is not eliminated. This radiation exchange is small in magnitude (i.e., does not strongly affect the values of ${Q}_{{{{{{\rm{d}}}}}}}$ or ${Q}_{{{{{{\rm{s}}}}}}}$ in the ON state), but is essential in determining the amplification parameter $\beta$. Figure 3e shows that the transistor achieves ON-state amplification values ranging from $\beta=8{-}31$ for $\Delta {T}_{{{{{{\rm{sd}}}}}}}$ between ${4}\deg {{{{{\rm{C}}}}}}{-}{17}\deg {{{{{\rm{C}}}}}}$ at gate heat flow increases $\Delta {Q}_{{{{{{\rm{g}}}}}}}\, > \,0.05\,{{{{{\rm{W}}}}}}$. This increase in the gate heat flow arises because power must be applied to the gate to heat up the Gd foil above ${T}_{{{{{{\rm{off}}}}}}-{{{{{\rm{on}}}}}}}$, and the increased gate temperature leads to an increase in the radiative heat flow from the gate to the source and drain. As expected, the $\beta$ values in Fig. 3e are larger than $\Delta {Q}_{{{{{{\rm{d}}}}}}}/\Delta {Q}_{{{{{{\rm{in}}}}}}}$ values found in Fig. 3b because $\Delta {Q}_{{{{{{\rm{g}}}}}}}\, < \,\Delta {Q}_{{{{{{\rm{in}}}}}}}$ due to thermal losses from the heater. The measured amplifications in the OFF state are much smaller in magnitude (e.g. $\beta={-}0.09$at $\Delta {Q}_{{{{{{\rm{g}}}}}}}=0.047\,{{{{{\rm{W}}}}}}$ for $\Delta {T}_{{{{{{\rm{sd}}}}}}}={7}\deg {{{{{\rm{C}}}}}}$), in agreement with the expectations for linear systems discussed in Supplementary Note 4. The error bars in the OFF state $\beta$ measurements in Fig. 3e originate from the uncertainties in measuring small $\Delta {Q}_{{{{{{\rm{d}}}}}}}$ arising from the applied $\Delta {Q}_{{{{{{\rm{g}}}}}}}$ in the OFF state. In contrast, it is straightforward to accurately measure the large $\Delta {Q}_{{{{{{\rm{d}}}}}}}$ in the ON state and the associated ON-state error bars in $\beta$ are smaller than the data points. Like an electrical FET, $\beta$ is not a constant parameter for all ${Q}_{{{{{{\rm{g}}}}}}}$ of interest, because the magnetic thermal transistor is most clearly understood as a transconductance device rather than as a current amplifier (i.e., the source–drain heat flow is fundamentally controlled by ${T}_{{{{{{\rm{g}}}}}}}$ rather than ${Q}_{{{{{{\rm{g}}}}}}}$). In addition, the binary nature of the switching means that the magnetic transistor cannot serve as a linear thermal amplifier (i.e., an arbitrary $\Delta {Q}_{{{{{{\rm{g}}}}}}}(t)$ waveform input at the gate is not reproduced in an amplified $\Delta {Q}_{{{{{{\rm{d}}}}}}}(t)$ waveform at the drain, though the frequency and phase of the waveform could potentially be extracted from the output signal). The amplification observed in our experiment is limited by our choice of maximum source temperatures near ${80}\deg {{{{{\rm{C}}}}}}$, which we selected to avoid Nd demagnetization. Larger $\beta$ could in theory be achieved with larger $\Delta {T}_{{{{{{\rm{sd}}}}}}}$ (as ${\Delta Q}_{{{{{{\rm{d}}}}}}}$ scales linearly with $\Delta {T}_{{{{{{\rm{sd}}}}}}}$ for fixed $G$) or slightly larger ${T}_{\infty }$ (such that smaller gate input powers ${Q}_{{{{{{\rm{in}}}}}}}$are needed for ${T}_{{{{{{\rm{g}}}}}}}$ to exceed ${T}_{{{{{{\rm{off}}}}}}-{{{{{\rm{on}}}}}}}$).

**Fig. 4:**
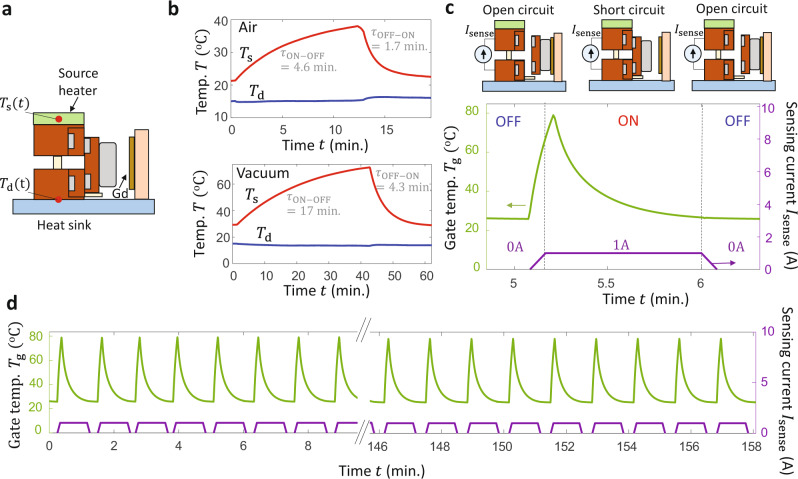
Transient transistor response and cycling. **a** To quantify the time response of the transistor, we apply a constant heating power to a source heater and record ${T}_{{{{{{\rm{s}}}}}}}(t)$ and ${T}_{{{{{{\rm{d}}}}}}}(t)$ after switching the transistor state via ${T}_{{{{{{\rm{g}}}}}}}$. **b** The characteristic transistor thermal time constants range from 1.7 to 4.6 min in air (top) and from 4.3 to 17 min in vacuum (bottom). The time response in the air is faster than in vacuum because the thermal contact resistances at the shuttle–source and shuttle–drain interfaces are smaller in the air than in a vacuum. **c** To evaluate the durability on cycling, we control ${T}_{{{{{{\rm{g}}}}}}}\left(t\right)$ (left), apply an electrical bias across the source to drain and use the measured sensing electrical current ${I}_{{{{{{\rm{sense}}}}}}}$ (right) to detect shuttle contact corresponding to ON and OFF states, as shown here for one cycle. **d** Continuous cycling measurements in the air show that the transistor switching is durable over more than 150 cycles.

Figure 4 investigates the transient response and thermal cycling of the transistor. Figure 4a illustrates our experimental setup, which eliminates the reference bars used in Fig. 2. The measured ${T}_{{{{{{\rm{s}}}}}}}(t)$ and ${T}_{{{{{{\rm{d}}}}}}}(t)$ in Fig. 4 are sensitive to the intrinsic time response of heat diffusion through the transistor itself, rather than being determined by the time response of the combined transistor/reference bar system. Figure 4b shows the thermal measurements in the air (top) and vacuum (bottom), in which we apply a constant input heating power to the source, heat sink the drain, and toggle ${T}_{{{{{{\rm{g}}}}}}}$ to cycle the transistor from ON–OFF–ON states. ${T}_{{{{{{\rm{d}}}}}}}$ is only weakly dependent on the transistor state because the drain is heat-sunk, while ${T}_{{{{{{\rm{s}}}}}}}$ increases in the transition to the OFF state and decreases in the transition to the ON state. We fit the time response of the transistor using a typical first-order exponential response and use these modeling fits to obtain characteristic ON–OFF and OFF–ON thermal time constants ${\tau }_{{{{{{\rm{ON}}}}}}-{{{{{\rm{OFF}}}}}}}$ and ${\tau }_{{{{{{\rm{OFF}}}}}}-{{{{{\rm{ON}}}}}}}$. In both vacuum and in air, ${\tau }_{{{{{{\rm{ON}}}}}}-{{{{{\rm{OFF}}}}}}}\, > \,{\tau }_{{{{{{\rm{OFF}}}}}}-{{{{{\rm{ON}}}}}}}$, because heat diffusion through the transistor is impeded in the low-conductance OFF state by the high thermal resistance of the polycarbonate support. In the air (top panel), ${\tau }_{{{{{{\rm{ON}}}}}}-{{{{{\rm{OFF}}}}}}}=4.6\,{\rm {min}}.$ and ${\tau }_{{{{{{\rm{OFF}}}}}}-{{{{{\rm{ON}}}}}}}=1.7\,{{{{{\rm{min}}}}}}.,$while in vacuum (bottom panel) ${\tau }_{{{{{{\rm{ON}}}}}}-{{{{{\rm{OFF}}}}}}}=17\,{\rm {min}}.$ and ${\tau }_{{{{{{\rm{OFF}}}}}}-{{{{{\rm{ON}}}}}}}=4.3\,{\rm {min}}.$ ${\tau }_{{{{{{\rm{OFF}}}}}}-{{{{{\rm{ON}}}}}}}$ is larger in a vacuum than in air because ${R}_{{{{{{\rm{contact}}}}}}}$ at the shuttle–source and shuttle–drain interface is larger in a vacuum than in air due to the lack of air pockets between the asperities on the rough surface. ${\tau }_{{{{{{\rm{ON}}}}}}-{{{{{\rm{OFF}}}}}}}$ is larger in a vacuum than in air because the parasitic OFF-state heat flow from source to drain through the air is eliminated in a vacuum. The thermal time constants for macroscopic (~centimeter scale) devices are orders of magnitude larger than the thermal time constants for micro/nanoscale systems because the thermal resistance and thermal capacitance of the device both scale with the thickness. For example, calculations for a proposed near-field thermal transistor using 200 nm thickness VO_2_ thin films^31^ predicted that the proposed transistor could dynamically modulate heat flows with <10 ms time response, though the device has not yet been experimentally investigated. In contrast, an experimentally demonstrated centimeter scale thermal switch using differential thermal expansion to make/break solid surface contact displayed ${\tau }_{{{{{{\rm{OFF}}}}}}-{{{{{\rm{ON}}}}}}}=12\,{{{{{\rm{min}}}}}}.,$which is of a similar order-of-magnitude to our demonstration^67^. These results indicate that ${\tau }_{{{{{{\rm{OFF}}}}}}-{{{{{\rm{ON}}}}}}}$ would be expected to decrease as the size of the device decreases and as ${G}_{{{{{{\rm{on}}}}}}}$ increases.

Figure 4c illustrates our electrical measurements used to test the repeatability of switching over extended thermal cycling. We apply an electrical bias between the source and drain and request an electrical sensing current of ${I}_{{{{{{\rm{sense}}}}}}}=1\,{{{{{\rm{A}}}}}}$ from a power supply. When the transistor is set ON with a large ${T}_{{{{{{\rm{g}}}}}}}$ (green), the shuttle is in contact with the source and drain and provides a pathway for both heat flow and electrical current. The power supply is able to provide this requested sensing current (purple) in the ON state because the device is electrically short-circuited. In the OFF state, however, the shuttle is not in contact with the source or drain and there is no pathway for electrical conduction. The power supply is unable to provide the requested electrical current in this open circuit scenario, and ${I}_{{{{{{\rm{sense}}}}}}}=0$ A. We use this electrical method to assess shuttle contact repeatability because ${I}_{{{{{{\rm{sense}}}}}}}$ is a rapid probe of switching that does not require the system to approach thermal steady-state, enabling more extended gate-temperature cycling measurements. We use the sensing current as a binary ON (=1 A)/OFF (=0 A) measurement, as annotated in Fig. 4c. The power supply that we use to measure the current has a resolution of 0.1 mA, which is larger than the current flowing through the dielectric polycarbonate dowel pin in the OFF state. Figure 4d shows that the switching is repeatable over >150 thermal switching cycles. In these measurements, we control ${T}_{{{{{{\rm{g}}}}}}}(t)$ (green) and record ${I}_{{{{{{\rm{sense}}}}}}}(t)$ (purple) through the continual cycling, as shown here for $t=0$ to $10$min. and for $t=146$to $158\,{{{{{\rm{min}}}}}} .$ time windows. We performed additional cycles to test the device over >1000 cycles. On the 980th cycle, we noted that the shuttle was in a partial-ON state (i.e. the shuttle was in a tilted orientation that did not contact the source to drain) at ${T}_{{{{{{\rm{g}}}}}}}={47}\deg {{{{{\rm{C}}}}}}$; increasing ${T}_{{{{{{\rm{g}}}}}}}$ to ${54}\deg {{{{{\rm{C}}}}}}$ caused a full-ON state, and we observed successful (i.e., complete) switching for all additional cycles. The partial switching could arise due to partial demagnetization of the Nd magnets upon thermal cycling; future applications could potentially utilize higher-grade magnets that are rated for elevated temperatures. ${T}_{{{{{{\rm{on}}}}}}-{{{{{\rm{off}}}}}}}$ and ${T}_{{{{{{\rm{off}}}}}}-{{{{{\rm{on}}}}}}}$ are both larger than in Figs. 4d and 2d due to an increased gap size between the shuttle and gate of 2.6 mm in the cycling measurements, leading to ${T}_{{{{{{\rm{on}}}}}}-{{{{{\rm{off}}}}}}}={27}\,\deg {{{{{\rm{C}}}}}}$ in Fig. 4d. The transistor did not fail over the >1000 cycles considered here, indicating that the switching performance is sufficiently repeatable for the laboratory-scale experiments and proof-of-concept applications discussed below.

### Proof-of-concept transistor circuits

**Fig. 5:**
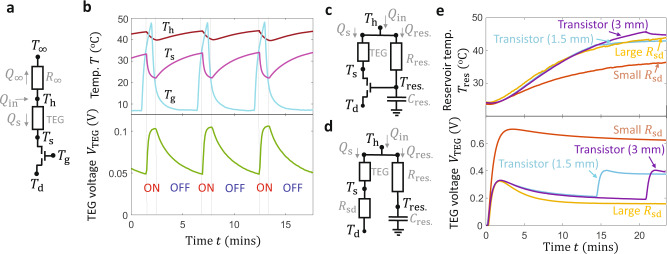
Thermal transistor circuit demonstrations. **a** Active heat routing thermal circuit and **b** experimental demonstration using a thermoelectric generator (TEG). When the transistor is set OFF using a low ${T}_{{{{{{\rm{g}}}}}}}$ (cyan, top), the low transistor ${G}_{{{{{{\rm{off}}}}}}}$ leads to a small TEG temperature difference (${T}_{{{{{{\rm{h}}}}}}}$ − ${T}_{{{{{{\rm{s}}}}}}}$) (brown and magenta, top) and small open-circuit TEG voltage ${V}_{{{{{{\rm{TEG}}}}}}}=0.05\,{{{{{\rm{V}}}}}}$ (bottom). When the transistor is set ON using a high ${T}_{{{{{{\rm{g}}}}}}}$, more of the input power flows through the transistor, leading to larger $({T}_{{{{{{\rm{h}}}}}}}-{T}_{{{{{{\rm{s}}}}}}})$ and larger ${V}_{{{{{{\rm{TEG}}}}}}}=0.1\,{{{{{\rm{V}}}}}}$. **c** Passive heat routing thermal circuit, **d** comparative thermal circuit using a thermal resistor ${R}_{{{{{{\rm{sd}}}}}}}$ instead of the transistor, and **e** experimental demonstration. The transistor thermal circuit in **c** directs the input heat flow ${Q}_{{{{{{\rm{in}}}}}}}$ to a thermal reservoir when the reservoir temperature ${T}_{{{{{{\rm{res}}}}}}}$ is low, while redirecting ${Q}_{{{{{{\rm{in}}}}}}}$ to pass through a TEG and generate power when ${T}_{{{{{{\rm{res}}}}}}}$ is high. **e** The transistor allows the system to simultaneously achieve large steady-state ${T}_{{{{{{\rm{res}}}}}}}\, > \,{40}\,^{{{{{{\rm{o}}}}}}}{{{{{\rm{C}}}}}}$ (top, cyan) and large steady-state ${V}_{{{{{{\rm{TEG}}}}}}}\, > \,0.4\,{{{{{\rm{V}}}}}}$ (bottom, cyan), combining beneficial aspects of the small-${R}_{{{{{{\rm{sd}}}}}}}$ (red) and large-${R}_{{{{{{\rm{sd}}}}}}}$ (yellow) cases. The transition switching temperature ${T}_{{{{{{\rm{off}}}}}}-{{{{{\rm{on}}}}}}}$ controls the steady-state reservoir temperature, and can be increased from ${41}\deg{{{{{\rm{C}}}}}}$ to ${46}\deg{{{{{\rm{C}}}}}}$ by increasing the gap size between the shuttle and the gate terminal from 1.5 mm (cyan) to 3 mm (purple).

Figure 5 shows that the magnetic thermal transistor enables thermal circuits for heat flow routing. Figure 5a illustrates an active routing circuit that thermally modulates the electrical output of a thermoelectric generator (TEG) used for energy scavenging. The gate temperature can be set above ${T}_{{{{{{\rm{off}}}}}}-{{{{{\rm{on}}}}}}}$to drive a large heat flow through the TEG only when the electrical power output is desired, reducing the need for auxiliary electrical controls or electrical storage during low-usage periods. In the thermal circuit representation of Fig. 5a, the input heat flow ${Q}_{{{{{{\rm{in}}}}}}}$ splits between a component ${Q}_{{{{{{\rm{s}}}}}}}$ that flows into the TEG and the transistor and a component ${Q}_{\infty }$ that flows to the ambient at ${T}_{\infty }$. In the low-${T}_{{{{{{\rm{g}}}}}}}$ case where the thermal transistor is OFF, the series thermal resistance $({R}_{{{{{{\rm{TEG}}}}}}}+{G}_{{{{{{\rm{off}}}}}}}^{-1})$ is relatively large; here, ${R}_{{{{{{\rm{TEG}}}}}}}$ is the effective thermal resistance of the TEG. For this demonstration we operate the TEG in open-circuit, meaning that the heat flows into and out of the TEG are identical and the thermal resistance representation of the TEG in Fig. 5a is accurate. Because the heat flow through the TEG is small when ${T}_{{{{{{\rm{g}}}}}}}\, < \,{T}_{{{{{{\rm{off}}}}}}-{{{{{\rm{on}}}}}}}$ and the transistor is OFF, the temperature difference (${T}_{{{{{{\rm{h}}}}}}}-{T}_{{{{{{\rm{s}}}}}}}$) and output open-circuit TEG voltage ${V}_{{{{{{\rm{TEG}}}}}}}$ are also relatively small, limiting the power output of the TEG that would be delivered to an electrical load (not included here). However, in the ${T}_{{{{{{\rm{g}}}}}}}\, > \,{T}_{{{{{{\rm{off}}}}}}-{{{{{\rm{on}}}}}}}$ case where the transistor is ON, ${Q}_{{{{{{\rm{s}}}}}}}$ and the TEG temperature difference $({T}_{{{{{{\rm{h}}}}}}}-{T}_{{{{{{\rm{s}}}}}}})$ are relatively large because ${T}_{{{{{{\rm{s}}}}}}}$ is pulled closer to ${T}_{{{{{{\rm{d}}}}}}}$ by the low transistor thermal resistance ${G}_{{{{{{\rm{on}}}}}}}^{-1}$. The potential electrical output power of the TEG would be larger compared to the low-${T}_{{{{{{\rm{g}}}}}}}$ state because a larger fraction of ${Q}_{{{{{{\rm{in}}}}}}}$ flows through the TEG as compared to the heat loss to the surroundings.

Figure 5b shows our measurements in air of the active heat flow routing circuit in Fig. 5a. We use a Peltier device to control ${T}_{{{{{{\rm{g}}}}}}}(t)$ (top panel, cyan) and set the transistor state. We then measure ${T}_{{{{{{\rm{s}}}}}}}(t)$, ${T}_{{{{{{\rm{h}}}}}}}(t)$, and ${V}_{{{{{{\rm{TEG}}}}}}}(t)$ in response to the applied ${Q}_{{{{{{\rm{in}}}}}}}$. When ${{T}}_{{{{{{\rm{g}}}}}}}={7}\deg {{{{{\rm{C}}}}}}$ and the transistor is OFF, the temperature difference (${T}_{{{{{{\rm{h}}}}}}}$-${T}_{{{{{{\rm{s}}}}}}}$) decreases to ${10}\deg {{{{{\rm{C}}}}}}$ and ${V}_{{{{{{\rm{TEG}}}}}}}$ is a fairly small 0.05 V, indicating that a relatively small fraction of ${Q}_{{{{{{\rm{in}}}}}}}$ is flowing through the transistor and thermoelectric due to the small transistor conductance ${G}_{{{{{{\rm{off}}}}}}}$. In our measurements, ${R}_{{{{{{\rm{TEG}}}}}}}\cong 2.9\,{{{{{\rm{K}}}}}}/{{{{{\rm{W}}}}}}$ and ${R}_{\infty }\cong 0.08\,{{{{{\rm{K}}}}}}/{{{{{\rm{W}}}}}}$, meaning that only 3% of the input power flows through the TEG when the transistor is OFF. When ${{T}}_{{{{{{\rm{g}}}}}}}={47}\deg {{{{{\rm{C}}}}}}$ and the transistor is ON, the large transistor ${G}_{{{{{{\rm{on}}}}}}}$ induces a larger heat flow through TEG, leading to larger TEG temperature difference $({T}_{{{{{{\rm{h}}}}}}}-{T}_{{{{{{\rm{s}}}}}}})={17.5}\deg {{{{{\rm{C}}}}}}$and open-circuit voltages ${V}_{{{{{{\rm{TEG}}}}}}}\, > \,0.1\,{{{{{\rm{V}}}}}}$. This demonstration shows that increasing ${T}_{{{{{{\rm{g}}}}}}}$ above the switching temperature drives ${T}_{s}$ towards ${T}_{{{{{{\rm{d}}}}}}}$ and controls the relative fraction of heat flow through the TEG as compared to the heat flow to the ambient. Though the ${T}_{{{{{{\rm{g}}}}}}}$ active tuning provides flexibility and user-dictated heat flow control, the downside of using this active thermal circuit is that power is consumed to control ${T}_{{{{{{\rm{g}}}}}}}$. The passive circuit discussed below overcomes this issue by eliminating the need for electrical power consumption to control ${T}_{{{{{{\rm{g}}}}}}}.$

Figure 5c illustrates a passive heat routing circuit that builds upon the basic elements of the active circuit shown in Fig. 5a. In the passive circuit of Fig. 5c, the transistor gate is mounted on a thermal reservoir with a temperature ${T}_{{{{{{\rm{res}}}}}}}$ and thermal capacitance ${C}_{{{{{{\rm{res}}}}}}}$. This reservoir is connected to the heated element by a thermal resistance ${R}_{{{{{{\rm{res}}}}}}}$, and an input heating power ${Q}_{{{{{{\rm{in}}}}}}}$ can flow into the thermal reservoir or into a parallel heat flow pathway through the TEG and transistor, as in the active circuit of Fig. 5a. This passive thermal circuit of Fig. 5c could be used in combined heat and power systems, as the circuit automatically switches from heating the thermal reservoir (which could represent, for example, a domestic hot water supply) to generating power via the TEG as the reservoir temperature increases above ${T}_{{{{{{\rm{off}}}}}}-{{{{{\rm{on}}}}}}}$. First, consider the case where ${T}_{{{{{{\rm{res}}}}}}}\, < \,{T}_{{{{{{\rm{off}}}}}}-{{{{{\rm{on}}}}}}}$. Because the transistor is OFF, there is a large thermal resistance to heat flow through the TEG and the input heat flow ${Q}_{{{{{{\rm{in}}}}}}}$ is routed to the thermal reservoir. The TEG generates a low output power during this initial portion of the cycle. Once ${T}_{{{{{{\rm{res}}}}}}}$ has been heated above ${T}_{{{{{{\rm{off}}}}}}-{{{{{\rm{on}}}}}}}$, the transistor turns ON and a larger fraction of the input power flows through the TEG to generate output electrical power. During this ON-state, the heat flow into the reservoir ${Q}_{{{{{{\rm{res}}}}}}.}$ is reduced, which passively regulates ${T}_{{{{{{\rm{res}}}}}}}$ near the switching temperature while also enabling power generation via a larger ${Q}_{{{{{{\rm{s}}}}}}}.$ This passive transistor circuit is advantageous compared to a traditional linear thermal circuit shown in Fig. 5d, in which the transistor is replaced with a resistor ${R}_{{{{{{\rm{sd}}}}}}}$. If ${R}_{{{{{{\rm{sd}}}}}}}$ is large, the comparative circuit routes heat to the thermal reservoir but does not generate a large output power because ${Q}_{{{{{{\rm{s}}}}}}}$ is relatively small. If ${R}_{{{{{{\rm{sd}}}}}}}$ is small, the TEG output voltage is large but the steady-state reservoir temperature is relatively low because the input heat flow is routed to the TEG for all $t$.

Figure 5e shows our measurements confirming that the passive transistor circuit of Fig. 5c with two different transistor source–drain gap sizes (cyan and purple lines) can combine beneficial aspects of the comparative small-${R}_{{{{{{\rm{sd}}}}}}}$ (red) and large-${R}_{{{{{{\rm{sd}}}}}}}$ (yellow) linear circuits of Fig. 5d. In all cases we apply similar heating conditions and measure ${T}_{{{{{{\rm{res}}}}}}}(t)$ (top) and ${V}_{{{{{{\rm{TEG}}}}}}}(t)$ (bottom) while the drain is kept at ${T}_{{{{{{\rm{d}}}}}}}={20}\deg {{{{{\rm{C}}}}}}$. During the initial heating at times $t\, < \,12\,{{{{{\rm{min}}}}}}.$, both transistors are OFF and the ${T}_{{{{{{\rm{h}}}}}}}$ and ${V}_{{{{{{\rm{TEG}}}}}}}$ measurements of the two transistor circuits are similar to that of the large-${R}_{{{{{{\rm{sd}}}}}}}$ case, indicating that heat is preferentially flowing to the reservoir. The transistor with a gap size of $1.5\,{{{{{\rm{mm}}}}}}$ (cyan) switches ON once ${T}_{{{{{{\rm{res}}}}}}}={41}\deg {{{{{\rm{C}}}}}}$. In this ON state, the heat flow through the TEG ${Q}_{{{{{{\rm{s}}}}}}}$ begins to increase. This increased heat flow leads to an enhanced steady-state ${V}_{{{{{{\rm{TEG}}}}}}}=0.4\,{{{{{\rm{V}}}}}}$ for the transistor as compared to ${V}_{{{{{{\rm{TEG}}}}}}}\, < \,0.2\,{{{{{\rm{V}}}}}}$ for the large-${R}_{{{{{{\rm{sd}}}}}}}$ case, demonstrating the passive heat routing capabilities of the transistor circuit. In contrast, the small-${R}_{{{{{{\rm{sd}}}}}}}$ (red line in Fig. 5e) scenario displays the highest steady-state output voltage ${V}_{{{{{{\rm{TEG}}}}}}}\, > \,0.6\,{{{{{\rm{V}}}}}}$ for all times $> 2\,{{{{{\rm{min}}}}}}.$ because there is a large heat flow through the TEG in this case; however, the reservoir temperature is ${7}\deg {{{{{\rm{C}}}}}}$lower for the small-${R}_{{{{{{\rm{sd}}}}}}}$scenario as compared to the 1.5 mm gap size transistor scenario (cyan), showing that small source-drain resistances are undesirable for the purpose of heating the reservoir. In all four cases, the voltages increase at short times before reaching a maximum and decreasing with increasing time. We attribute this maximum in the voltage to the effects of heat diffusion in the TEG, which leads to slightly larger transient temperature differences as compared to steady-state.

Figure 5e shows that the transistor successfully regulates the temperature of the reservoir near ${T}_{{{{{{\rm{off}}}}}}-{{{{{\rm{on}}}}}}}$ while also providing a small thermal resistance for power generation. The regulation temperature of the reservoir can be tuned by controlling the shuttle-gate gap size; comparing the 1.5 mm gap transistor (cyan) and the 3 mm gap transistor (purple) in Fig. 5e shows that increasing the gap size increases ${T}_{{{{{{\rm{off}}}}}}-{{{{{\rm{on}}}}}}}$ from ${41}\deg {{{{{\rm{C}}}}}}$ to ${46}\deg {{{{{\rm{C}}}}}}$, which also delays the time at which ${V}_{{{{{{\rm{TEG}}}}}}}$ increases above $0.4$V from 15 to 22 min. Note that these values of ${T}_{{{{{{\rm{off}}}}}}-{{{{{\rm{on}}}}}}}$ are larger than the value of ${27}\deg {{{{{\rm{C}}}}}}$ found in Fig. 2d due to the larger gap sizes used in Fig. 5e; we chose these larger gap sizes to show that the reservoir temperature could be regulated well above room temperature. Overall, this demonstration in Fig. 5e shows that the passive transistor action can be used for heat flow routing and thermal regulation without external power consumption to control the gate.

**Fig. 6:**
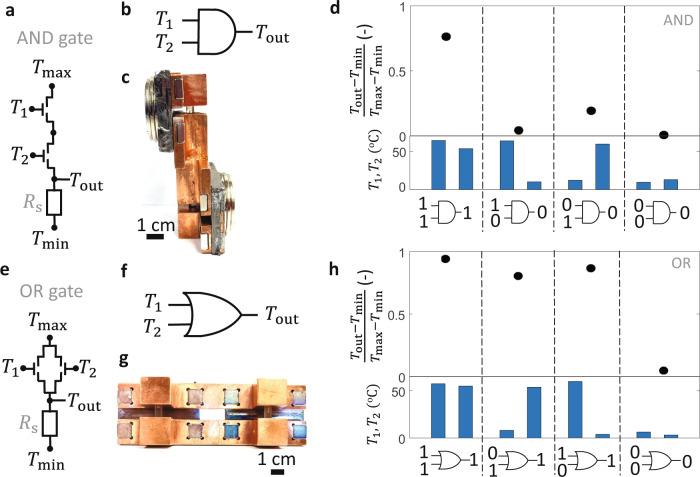
Thermal logic gate demonstration. **a** Thermal circuit schematic, **b** logic gate representation, and **c** image of thermal AND gate using two transistors thermally in series between a maximum supply temperature ${T}_{{{\max }}}$ and minimum supply temperature ${T}_{\min }$. The input temperatures ${T}_{1}$ and ${T}_{2}$are connected to the gate terminals, and the output temperature ${T}_{{{{{{\rm{out}}}}}}}$ is separated from ${T}_{\min }$ by a series thermal resistor ${R}_{{{{{{\rm{s}}}}}}}$. **d** Measured AND gate truth table. The output normalized temperature for the four cases is a Boolean 1 (HIGH) only if ${T}_{1}$ and ${T}_{2}$ are both larger than ${T}_{{{{{{\rm{off}}}}}}-{{{{{\rm{on}}}}}}}$, while the output temperature is a Boolean 0 (LOW) otherwise. **e** Thermal circuit schematic, **f** logic gate representation and **g** image of thermal OR gate consisting of two transistors thermally in parallel. **h** The measured OR gate truth table showing that the thermal output is HIGH if either ${T}_{1}$ or ${T}_{2}$ is larger than ${T}_{{{{{{\rm{off}}}}}}-{{{{{\rm{on}}}}}}}$ and LOW otherwise.

Figure 6 shows that the thermal transistors can also be used to create thermal logic gates, which are Boolean devices in which the combination of two input temperatures $({T}_{1},{T}_{2})$ dictates whether the output temperature ${T}_{{{{{{\rm{out}}}}}}}$ is driven towards a maximum supply temperature ${T}_{{{\max }}}$ or a minimum supply temperature ${T}_{\min }$. Thermal logic devices could be useful for thermal sensing with minimal thermal readout requirements. For example, a single output temperature from a thermal circuit consisting of thermal OR gates could be used to detect whether any of $N$ the input temperatures (${T}_{1}$, ${T}_{2}$,…., ${T}_{{{{{{\rm{N}}}}}}}$) is above the switching temperature, as illustrated in Supplementary Figure 4 for $N=8$. Like mechanical^68^, fluidic^69^, and textile^70^ logic elements, thermal logic devices could potentially be applied for computation and sensing without requiring electrical power consumption, if a naturally occurring thermal gradient exists to establish ${T}_{\min }$ and ${T}_{{{\max }}}$. Although nanoscale thermal–mechanical logic elements leveraging thermal expansion and gap-size-dependent thermal radiation have been demonstrated^71^, macroscopic thermal logic gates have not been reported.

Figure 6a shows the thermal circuit for our thermal AND gate represented in Fig. 6b. The two input temperatures ${T}_{1}$ and ${T}_{2}$ are each connected to a transistor gate temperature, and the transistor source–drain connections are placed thermally in series. A thermal resistor ${R}_{{{{{{\rm{s}}}}}}}$ separates the drain of a transistor from ${T}_{\min }$, allowing the output temperature ${T}_{{{{{{\rm{out}}}}}}}$ to swing between a HIGH value (Boolean 1) near ${T}_{{{\max }}}$ if both transistors are ON and a LOW value (Boolean 0) near ${T}_{\min }$ if either transistor is OFF. A thermal transistor with a large switching ratio is needed for this application because achieving the output swing requires ${R}_{{{{{{\rm{s}}}}}}}\, \gg \,{2G}_{{{{{{\rm{on}}}}}}}^{-1}$ to pull the output HIGH in the Boolean 1 state and ${R}_{{{{{{\rm{s}}}}}}}\, \ll \, {G}_{{{{{{\rm{off}}}}}}}^{-1}$ to pull the output LOW in the Boolean 0 state; these requirements can only be satisfied if ${G}_{{{{{{\rm{on}}}}}}}/{G}_{{{{{{\rm{off}}}}}}}$ is large compared to unity, as in our demonstration. We used a series resistance with ${R}_{{{{{{\rm{s}}}}}}}=33\frac{{{{{{\rm{K}}}}}}}{{{{{{\rm{W}}}}}}}$ to satisfy these requirements.

Figure 6c is an optical image of our fabricated thermal AND gate. The two shuttles are located on opposite sides of the main transistor body to reduce the potential impact of thermal and magnetic interference between the two input temperatures. Graphite foils are wrapped around the shuttles as thermal interface materials. We use a thermocouple placed between the second transistor and the series resistor (not shown) to measure ${T}_{{{{{{\rm{out}}}}}}}.$ Figure 6d shows our measurements in air of the dimensionless output temperature $\frac{{T}_{{{{{{\rm{out}}}}}}}-{T}_{\min }}{{T}_{{{\max }}}-{T}_{\min }}$ (top panel) for different input temperature combinations ${T}_{1}$ and ${T}_{2}$ (bottom panel). This thermal truth table displays the expected AND gate performance in which the dimensionless output temperature is HIGH (0.76) if ${T}_{1}$ and ${T}_{2}$ are both set above ${T}_{{{{{{\rm{off}}}}}}-{{{{{\rm{on}}}}}}}$ and the dimensionless output temperature is LOW (ranging from 0.006 to 0.2) if one or both of the input temperatures are below ${T}_{{{{{{\rm{on}}}}}}-{{{{{\rm{off}}}}}}}$. The (1,0) and (0,1) cases do not have identical analog output temperatures, which we attribute to differences in the contact interfaces and contact pressures between the two shuttles and their respective transistor bodies. The HIGH and LOW analog output values could be further improved by increasing the thermal contact conductances and switch ratio of the device. Using a vacuum environment would reduce the parasitic losses to the surroundings, though the vacuum would also decrease ${G}_{{{{{{\rm{on}}}}}}}$ due to the enhanced ${R}_{{{{{{\rm{contact}}}}}}}$, which is undesirable. Lastly, we note that minor modifications to the circuit topology of the AND gate (i.e. switching the locations of ${T}_{\min }$ and ${T}_{{{\max }}}$) result in a thermal NAND gate, as illustrated in the thermal circuit of Supplementary Fig. 5a.

Similarly, Fig. 6e–g shows the thermal circuit, logic gate symbol, and fabrication of a thermal OR gate, respectively. In the OR gate, two transistors are arranged thermally in parallel, such that the two transistors are connected to the same source and drain temperature. The input temperatures ${T}_{1}$and ${T}_{2}$ also correspond to the temperatures of the two transistor gates, and the output temperature is located at the shared transistor drain, which is separated from ${T}_{\min }$ by the series resistor ${R}_{{{{{{\rm{s}}}}}}}$. If either or both of ${T}_{1}$ or ${T}_{2}$ are set above ${T}_{{{{{{\rm{off}}}}}}-{{{{{\rm{on}}}}}}}$, ${T}_{{{{{{\rm{out}}}}}}}$ is pulled HIGH towards ${T}_{{{\max }}}$, while if ${T}_{1}$ and ${T}_{2}$ are both below ${T}_{{{{{{\rm{on}}}}}}-{{{{{\rm{off}}}}}}}$, ${T}_{{{{{{\rm{out}}}}}}}$ is LOW. This OR gate behavior is observed when ${R}_{{{{{\rm{{s}}}}}}}\, \gg \, {G}_{{{{{{\rm{on}}}}}}}^{-1}/2$ and ${R}_{{{{{{\rm{s}}}}}}}\, \ll \,{G}_{{{{{{\rm{off}}}}}}}^{-1}$. Figure 6h shows our measurements of the OR gate performance, and confirms that the dimensionless output temperature is HIGH (ranging from 0.80 to 0.94) if ${T}_{1}$ and/or ${T}_{2}$ are larger than ${T}_{{{{{{\rm{off}}}}}}-{{{{{\rm{on}}}}}}}$, and that the dimensionless output temperature is LOW (0.05) if ${T}_{1}$ and ${T}_{2}$ are both smaller than ${T}_{{{{{{\rm{on}}}}}}-{{{{{\rm{off}}}}}}}$. Supplementary Fig. 5b shows that a similar NOR gate can be constructed from this OR gate by switching the location of ${T}_{\min }$and ${T}_{{{\max }}}$. Thus, the thermal transistor can be used to create four two-input one-output Boolean logic elements AND, OR, NAND, and NOR. Moreover, in Supplementary Fig. 8 we experimentally demonstrate that the transistor can also be combined with a thermal resistor to construct a one-input one-output NOT logic gate (i.e., Input 1 = Output 0, Input 0 = Output 1).

## Discussion

Our thermal transistor uses ${T}_{{{{{{\rm{g}}}}}}}$-dependent magnetic forces to make and break thermal contact between the source and drain. Other ${T}_{{{{{{\rm{g}}}}}}}$-dependent forces could also be used in similar contact-based thermal transistors; for example, well-established passive thermal switch mechanisms based on thermal expansion of waxes^67^, shape-memory alloys^16,64^, or the differential coefficient of thermal expansion devices^72^ could plausibly be adapted into three-terminal transistor geometries. A potential advantage of the magnetic mechanism as compared to thermal expansion is that the governing magnetic forces act at a distance without any intermediary matter, leading to relatively low gate heat flow leakage in both the ON and OFF states arising only from thermal radiation in a vacuum. In contrast, thermal expansion transistors may need to consider the conduction heat flow from the gate to the drain and source, as there must be a proximal material contact to actuate the motion of the shuttle via thermal expansion. The magnetic transistor also uses relatively large gap sizes (here 1–3 mm) compared to thermal expansion switches (e.g. 0.05 mm^72^), which would assist in reducing the gate heat leakage via conduction through the air between the gate and the shuttle for applications in ambient conditions. The gap size-dependent response of the magnetic transistor is also useful in fine-tuning the gate switching temperatures and thermal deadband; choosing a ferromagnetic gate material with a different ${T}_{{{{{{\rm{Curie}}}}}}}$ would enable switching temperatures that are well above or below room temperature. Though our device has ~cm length scales, future work could explore transistor variants with different characteristic dimensions. Macroscopic applications in energy harvesting or building thermal control may require larger transistors that can sustain high ON-state heat flows. Increasing the ON state magnetic contact pressures and/or surface area of the shuttle–source drain contact would assist in increasing ${G}_{{{{{{\rm{on}}}}}}}$ for these applications, and finite-element models could be used to optimize the design. At the other end of the spectrum, miniaturization would be desired for logic applications involving a large number of transistors, although the gating mechanism might be challenging to adapt at a small scale due to the effects of stiction and/or magnetic interference between separate transistors.

The magnetic thermal transistor is straightforward to fabricate at the benchtop scale using commercially available materials, making the device an appealing option for thermal scientists to explore thermal circuits and potential applications of three-terminal thermal transistors. Achieving the ultimate impact of thermal transistors in thermal systems will require further research exploring miniaturization, scalable manufacturing methods, cost reduction, durability enhancement, and magnetic shielding/confinement. In particular, the durability of the contact surface and the copper terminals would need to be studied further, particularly in applications under harsh environments. The main envisioned modes of degradation are tearing/wear of the thermal interface material (TIM) on the shuttle, fouling/oxidation of copper surfaces, and demagnetization of the neodymium magnets. If surface oxidation is a concern, aluminum could be used instead of copper, though the pristine ${G}_{{{{{{\rm{on}}}}}}}$ would likely decrease due to the lower thermal conductivity of aluminum compared to copper. Removing the TIM and using bare metal–metal contacts would also likely reduce ${G}_{{{{{{\rm{on}}}}}}}$ unless ultrafine surface roughnesses can be achieved. To prevent the demagnetization of the permanent magnets, the device should be operated in the rated temperature range. High-temperature magnets are commercially available, albeit at a higher cost compared to the Nd alloy magnets used here. Though commercially appealing applications of thermal transistors would be required to drive a thermal Moore’s law scaling of cost, performance, and size for thermal transistors, the evolution of electrical transistors from their first lab-scale demonstrations to the modern semiconductor industry’s global impact provides a tantalizing motivation for further research exploring a range of thermal transistor mechanisms and applications.

In summary, we demonstrated thermal switching and thermal amplification of the source–drain heat flow by controlling the gate temperature of a three-terminal magnetic thermal transistor. The switching is controlled by heating the gate terminal in the vicinity of the Curie temperature of gadolinium, a material that undergoes a ferromagnetic-to-paramagnetic phase transition near room temperature. The transistor’s negative differential thermal resistance arises from the large variations in the source-to-drain heat flow when the gate temperature is toggled above or below the switching temperature to passively control the steady-state location of the thermally conducting shuttle. We measure switching ratios of $$109\pm 44$$ and amplification ratios $$ > 30$$ with thermal deadbands as small as $${7}\deg {{{{{\rm{C}}}}}}$$, and use electrical sensing measurements to demonstrate that the switching is repeatable on thermal cycling of the gate. The switching properties of the transistor can be leveraged to actively or passively route heat flows in a combined thermal storage/power generation thermal circuit, or to process thermal information in thermal AND/OR logic gates. The concept and demonstration established by this work will motivate further exploration of thermal transistor mechanisms and potential applications for thermal sensing, thermal control, and thermal management.

## Methods

### Device fabrication

We assembled the thermal transistor using commercially available materials. The source terminal, the drain terminal, and the shuttle were machined from a 110 copper alloy bar (McMaster, 89275K41). The gadolinium foil mounted on the gate terminal has dimensions of 25 by 25 by 1 mm^3^ (693723-1EA, Sigma-Aldrich). A polycarbonate dowel pin (92078A217, McMaster) is used to provide mechanical support between the source and drain terminals. Permanent magnets used in the device include eight square neodymium-iron-boron magnets with dimensions of 6.3 by 6.3 by 2.5 mm^3^ (NSN0610, Magcraft), two-disc neodymium–iron–boron magnets of diameter 25 mm and thickness 1.6 mm (NSN0749 magnets, Magcraft), and one nickel-plated neodymium disc magnet of diameter 25 mm and thickness 1.6 mm (5862K153, McMaster). For simplicity, we refer to all permanent magnets as Nd throughout the text. We polished thermally contacting surfaces on the source, drain, and shuttle over a range of sandpaper grits from 220-grit up to 10,000-grit to create a mirror finish and reduce the thermal contact resistances associated with surface asperities. To further improve the contact resistance, we applied a 25 ${{{{{\rm{\mu }}}}}}{{{{{\rm{m}}}}}}$thick graphite sheet (1334N1, McMaster) on the shuttle as a thermal interface material; the sheet is not included in the images of Fig. 1 to better display the Nd magnets embedded in the shuttle. The AND and OR gates were fabricated using the same copper stock, magnets, and gadolinium foils as the individual transistor used in the thermal conductance measurements.

We chose the cross-sectional area of the source and drain to match the reference bar cross-sectional area of 1.2 by 1.2 cm, promoting one-dimensional heat transfer in the reference bar. The area of shuttle contact with the source and drain was selected to be a relatively large 2.7 by 3.8 cm because maximizing this contact area reduces the total thermal contact resistance. The location of the square magnets inset in the source, drain, and the shuttle was selected to minimize the distance between the source/drain magnets and the shuttle magnets, which assists in enhancing the contact pressures and reducing ${R}_{{{{{{\rm{contact}}}}}}}$. The primary consideration for the geometry of the disc magnets placed on the shuttle and the gap size between gate and shuttle magnets aimed to maximize the interaction strength with the gate terminal, providing the device actuation. Small gap sizes are generally desirable for reduced thermal deadband, but we also observed that very small gap sizes (e.g. <1 mm) could result in a tilted shuttle location that was neither fully ON nor fully OFF, motivating our use of ~1–3 mm gap sizes. To ensure that gravitational effects did not impede repeatable switching, we inserted an acrylic ledge (0.15 by 0.8 by 1.2 cm^3^) extending from the drain. The acrylic makes contact with the shuttle in the ON state but does not interact with the gate in either the ON state or the OFF state.

### Thermal measurements

All measurements were performed inside a chamber (ISO100-K, Kurt J. Lesker) either held at ambient pressure or placed under a high vacuum (~10^−5^ Pa). The reference bars were machined from paramagnetic stainless-steel stock (4539T17, McMaster) and measured 1.2 by 1.2 by 5 cm. Each reference bar has four thermocouple holes drilled with a spacing between thermocouples of 1 cm. Thermal paste (ARCTIC-MX4, Arctic) was used on the metal-on-metal contacts to minimize the contact thermal resistances between the reference bars and the source/drain terminals. Thermal paste was also applied to the thermocouple beads before insertion in the reference bars. In most of our measurements, Peltier modules (TEC1-12706, Geebat) were mounted on the gate terminal and above the top reference bar to control ${T}_{{{{{{\rm{g}}}}}}}$ and ${T}_{{{{{{\rm{s}}}}}}}$, respectively. Electrical power and control were provided using a d.c. power supply (Keithley 2100). A cold plate with a closed water loop is used to control ${T}_{{{{{{\rm{d}}}}}}}$ in experiments when a heat sink is required. We used thick-gauge thermocouples (TJ72-CASS-010G-2, OMEGA) inserted in our reference bar experiments to measure the temperature gradient and fine-gauge (0.005” diameter, CHAL-005, Omega) thermocouples for temperature measurements on surfaces. Fiberglass-reinforced aerogel (9590K21, McMaster) was used for thermal insulation for measurements in air.

We calculate the heat flow in our reference bar experiments as

1 $${Q}_{{{{{{\rm{d}}}}}}}={{A}_{{{{{{{\rm{rb}}}}}}}}\kappa }_{{{{{{{\rm{rb}}}}}}}}\frac{{{{{{{\rm{d}}}}}}T}}{{{{{{{\rm{d}}}}}}z}}-{Q}_{{{{{{{\rm{rad}}}}}}}},$$

where ${\kappa }_{{{{{{\rm{rb}}}}}}}=16\frac{{{{{{\rm{W}}}}}}}{{{{{{\rm{m}}}}}}.{{{{{\rm{K}}}}}}}$ is the thermal conductivity of our stainless-steel reference bars, ${A}_{{{{{{\rm{rb}}}}}}}$= 1.27 by 1.27 cm^2^ is the cross-sectional area of the reference bars, and $\frac{{dT}}{{dz}}$ is the temperature gradient along the lower reference bar extracted from the measured $T$ values, as discussed in Supplementary Note 2. The radiation heat transfer exchange from the warmer surroundings to the lower reference bar in Eq. (1) is computed as

2 $${Q}_{{{{{{{\rm{rad}}}}}}}}=\varepsilon \sigma {A}_{{{{{{\rm{s}}}}}}}({{T}_{{{\infty }}}}^{4}-{\bar{T}}^{4}),$$

where $\sigma$ is the Stefan–Boltzmann constant, $\varepsilon=0.2$is the emissivity of the stainless-steel reference bar, ${A}_{{{{{{\rm{s}}}}}}}=12\,{{{{{\rm{c}}}}}}{{{{{{\rm{m}}}}}}}^{2}$ is the reference bar surface area between the central thermocouple in the lower reference bar and the drain, ${T}_{\infty }={20}\deg {{{{{\rm{C}}}}}}$, and $\bar{T}$ is the average temperature measured by the thermocouples in the lower reference bar. Equation (2) is valid for an isothermal body exchanging heat with large blackbody surroundings. Due to the low ${Q}_{{{{{{\rm{d}}}}}}}$ in the OFF state, temperature gradients in the reference bar are minimal (with typical <${2}\deg {{{{{\rm{C}}}}}}$ temperature variation along the bar), satisfying the assumptions of Eq. (2). In the OFF state, ${Q}_{{{{{{\rm{rad}}}}}}}$ is typically ~20% of ${Q}_{{{{{{\rm{d}}}}}}}$, meaning that the radiation is non-negligible in the analysis. In the ON state, ${Q}_{{{{{{\rm{rad}}}}}}}$ is two orders of magnitude smaller than the measured ${Q}_{{{{{{\rm{d}}}}}}}$, meaning that radiation does not substantially influence the measurements. The source-drain temperature difference ${\Delta T}_{{{{{{\rm{sd}}}}}}}$is the temperature drop across the transistor itself and is found by extrapolating a linear fit to the reference bar temperatures. For example, the thermal conductance data shown in Fig. 2c is extracted from the measured thermocouple data in Fig. 2b using Eqs. (1) and (2) along with the extrapolated ${\Delta T}_{{{{{{\rm{sd}}}}}}}.$

### Amplification experiment

For the amplification experiment in Fig. 3, we modified the thermal apparatus used in Fig. 2a to incorporate a film heater (PLMLV-101/10, OMEGA) instead of a Peltier module to control ${T}_{{{{{{\rm{g}}}}}}}$. The transistor was initialized in the OFF state, and the input power to the film heater was gradually increased until the transistor switched to the ON state. This input heat flow ${Q}_{{{{{{\rm{in}}}}}}}$ was measured using the voltage and current supplied to the film heater. The gate heat flow ${Q}_{{{{{{\rm{g}}}}}}}$ is smaller than ${Q}_{{{{{{\rm{in}}}}}}}$ and was calculated using finite element method (FEM) thermal simulations that included the radiative exchange between the transistor elements and the blackbody surroundings at a temperature ${T}_{\infty }={21}\,\deg {{{{{\rm{C}}}}}}$. The emissivities of the gadolinium and neodymium were chosen to give a conservative estimate of the amplification ratio and are taken as ${\varepsilon }_{{{{{{\rm{Gd}}}}}}}=0.4$and ${\varepsilon }_{{{{{{\rm{Nd}}}}}}}=0.4$, respectively^73^. The distance between the gate and the shuttle is $2$ mm for our simulation. We modeled conduction through the transistor, radiation between the gate and the shuttle, and radiation from all surfaces to the surroundings. The measurement is performed in vacuum, so there is no conduction/convection losses to the surroundings. The FEM calculations are used to calculate the value of ${Q}_{{{{{{\rm{g}}}}}}}$ for a given set of source, drain, and gate temperatures. $\Delta {Q}_{{{{{{\rm{g}}}}}}}$ in Fig. 3e is the difference between ${Q}_{{{{{{\rm{g}}}}}}}$ at the measured ${T}_{{{{{{\rm{g}}}}}}}$ when heating is applied and ${Q}_{{{{{{\rm{g}}}}}}}$ when ${T}_{{{{{{\rm{g}}}}}}}={T}_{\infty }$ and no external heating is applied. This value of $\Delta {Q}_{{{{{{\rm{g}}}}}}}$ represents the increase in the heat flow from the gate into the source/drain of the transistor due to the enhanced gate temperature, and is positive for all values of ${T}_{{{{{{\rm{g}}}}}}}\, > \,{T}_{\infty }$ considered here. Because the gate heat flow ${Q}_{{{{{{\rm{g}}}}}}}$ is much smaller than ${Q}_{{{{{{\rm{s}}}}}}}$ in the ON state (as discussed in Fig. 3 above), we found that ${Q}_{{{{{{\rm{s}}}}}}}$ required to achieve a fixed $\Delta {T}_{{{{{{\rm{sd}}}}}}}$ does not depend strongly on ${T}_{{{{{{\rm{g}}}}}}}$ (or equivalently the power input to the gate). In the OFF state, however, variations in ${T}_{{{{{{\rm{g}}}}}}}$ do influence $\Delta {T}_{{{{{{\rm{sd}}}}}}}$ via the radiative heat transfer from the gate, meaning that the ${Q}_{{{{{{\rm{d}}}}}}}$ needed to achieve a fixed $\Delta {T}_{{{{{{\rm{sd}}}}}}}$ changes with ${T}_{{{{{{\rm{g}}}}}}}$ in the OFF state. For example, when we increased ${Q}_{{{{{{\rm{in}}}}}}}$ from 0 to 0.19 W in the OFF state of Fig. 3b, we found that ${\Delta T}_{{{{{{\rm{sd}}}}}}}$ increases by ${1.3}\deg {{{{{\rm{C}}}}}}$. We do not observe a measurable change in ${\Delta T}_{{{{{{\rm{sd}}}}}}}$ for a 0.13 W increase in ${Q}_{{{{{{\rm{in}}}}}}}$ from 0.3 to 0.43 W in the ON state.

### Time response experiment

Our time response experiments and electrical cycling measurements eliminated the stainless steel reference bars to focus on the intrinsic time constant of the transistor, as illustrated in Fig. 4a. We used the film heater from the amplification experiment to control ${T}_{{{{{{\rm{s}}}}}}}$, and use a $1\,{{{{{\rm{W}}}}}}$ input heating power. Fine-gauge thermocouples were used to measure all relevant temperatures. We calculate the OFF–ON time constant and the ON–OFF time constant by fitting an exponential function to the measured data. To understand the origin of the dominant time constants in the system, we calculated the one-dimensional thermal diffusion times ${\tau }_{{{{{{\rm{diff}}}}}}}={L}^{2}/4\alpha$ for each of the individual components, where $L$ is the thickness of the element and $\alpha$ is the thermal diffusivity. We carried out this analysis for the polycarbonate support, the copper shuttle, and the copper source/drain terminals. The computed time constant for the polycarbonate support (${\tau }_{{{{{{\rm{pc}}}}}}}=266\,{{{{{\rm{s}}}}}}$) is two orders of magnitude greater than the time constant for the other components (${\tau }_{{{{{{\rm{shuttle}}}}}}}=1.5\,{{{{{\rm{s}}}}}}$ and ${\tau }_{{{{{{\rm{terminal}}}}}}}=0.28\,{{{{{\rm{s}}}}}}$), indicating that ${\tau }_{{{{{{\rm{ON}}}}}}-{{{{{\rm{OFF}}}}}}}$ is limited by the diffusion through the polycarbonate support$.$ In the OFF–ON transition, the heat diffuses quickly through the source/drain terminals and through the shuttle itself as shown by the relatively small ${\tau }_{{{\rm{shuttle}}}}$ and ${\tau }_{{{{{{\rm{terminal}}}}}}}$ above. The limiting factor for the OFF–ON time response of the system arises from the thermal contact resistance between the shuttle and the source/drain terminals.

### Heat routing circuit demonstrations

Our active heat routing demonstration in Fig. 5a used a TEG in series with the transistor. The gate temperature was controlled using a Peltier module and a power supply, and we powered a film heater placed in series with the TEG with an electrical power of $1.7\,{{{{{\rm{W}}}}}}$. To determine ${R}_{{{{{{\rm{TEG}}}}}}}$and ${R}_{\infty }$ we used FEM combined with our experimental results. For the TEG, we measured ${R}_{{{{{{\rm{TEG}}}}}}}$ using our reference bar apparatus. In our FEM simulation, we included the transistor in series with the TEG in one branch and the copper and steel support for the other branch. We modeled radiation between all surfaces and the surroundings, convection between all surfaces and the surroundings, and conduction through all solid surfaces in contact. To find the convection coefficients for the surrounding air we used the standard Nusselt correlations for a vertical plate, horizontal plate, and vertical thin cylinder included in the FEM software package. We selected the temperature boundary conditions in the simulations to match the measured ${T}_{{{{{{\rm{h}}}}}}}$ and ${T}_{{{{{{\rm{d}}}}}}}$ in Fig. 5b. We used the steady-state FEM result to determine the heat flow splitting between the two branches and to find ${R}_{\infty }$.

The passive heat routing experiments in Fig. 5c and d incorporate a copper reservoir with capacitance ${C}_{{{{{{\rm{res}}}}}}}\cong 13\,{{{{{\rm{J}}}}}}/{{{{{\rm{K}}}}}}$ contacting the gadolinium foil and gate. A 1.7 mm-thick acrylic slab with thermal resistance ${R}_{{{{{{\rm{res}}}}}}}\cong 22\,{{{{{\rm{K}}}}}}/{{{{{\rm{W}}}}}}$ separates this copper reservoir from the heat source. In these passive heat routing experiments, the biasing power was provided by a Peltier module. For the comparative small-${R}_{{{{{{\rm{sd}}}}}}}$ case illustrated in Fig. 5d, we used a copper block with dimensions 38 by 38 by 27.5 mm^3^ in place of the transistor, achieving a low value of ${R}_{{{{{{\rm{sd}}}}}}}\cong 0.09\,{{{{{\rm{K}}}}}}/{{{{{\rm{W}}}}}}$. For the large-${R}_{{{{{{\rm{sd}}}}}}}$ comparative case, we used the transistor with the shuttle removed, such that the only heat transfer pathway from source to drain occurs via parasitic conduction or radiation. The estimated resistance for this shuttle-removed scenario is ${R}_{{{{{{\rm{sd}}}}}}}\cong 270\,{{{{{\rm{K}}}}}}/{{{{{\rm{W}}}}}}.$ Images of the components used in the passive heat routing experiment are shown in Supplementary Fig. 7.

### Finite-element thermal calculations

We performed thermal calculations using the commercial finite-element software COMSOL Multiphysics, as discussed in detail in Supplementary Note 1.

## Supplementary information

Supplementary Information

Description of Additional Supplementary Files

Supplementary Movie 1

## Data Availability

The data that support the findings of this study are available from the corresponding author upon request.

## Code availability

Finite element calculations are available from the corresponding author upon request.
