# Supplementary material for: A three-terminal magnetic thermal transistor
Source: Nat Commun. 2023 Jan 24;14:393. doi: 10.1038/s41467-023-36056-4 (PMC9873738; doi:10.1038/s41467-023-36056-4)
Supplement: Supplementary file 1 — Supplementary Information [file 41467_2023_36056_MOESM1_ESM.pdf]

Supplementary Information

A three-terminal magnetic thermal transistor

Lorenzo Castelli [1], Qing Zhu [1], Trevor J. Shimokusu [1], Geoff Wehmeyer [1+]

1 Mechanical Engineering, William Marsh Rice University, Houston TX, 77005

[+]Corresponding author, gpw1@rice.edu

Supplementary Note 1: Finite element modeling for ON and OFF state thermal conductance

In our thermal FEM calculations, we modeled the thermal transistor to extract R_{contact} in the ON state and to quantify the dominant modes of heat transfer in the OFF state. The geometry of the FEM calculations includes the reference bars, the source and drain terminals of the transistor, the shuttle, and the gadolinium foil on the gate terminal. We imposed constant-temperature boundary conditions on the exterior boundaries of the reference bars using temperatures recorded from experimental data. We use standard handbook values for the material properties. We modeled radiation between all surfaces and the blackbody surroundings using a gadolinium, neodymium, copper, and reference-bar emissivity $\varepsilon_{\mathrm{Gd}} = 0.4, \varepsilon_{\mathrm{Nd}} = 0.4,$ $\varepsilon_{\mathrm{Cu}} = 0.3,$ and $\varepsilon_{\mathrm{rb}} = 0.2,$ respectively. We modeled conduction through the transistor using handbook copper, reference bar, polycarbonate, and neodymium thermal conductivities of $k_{\mathrm{Cu}} = 400\,\frac{\mathrm{W}}{\mathrm{m.K}}$, $k_{\mathrm{rb}} = 16\,\frac{\mathrm{W}}{\mathrm{m.K}}$, $k_{\mathrm{pc}} = 0.2\,\frac{\mathrm{W}}{\mathrm{m.K}}$, and $k_{\mathrm{Nd}} = 11\,\frac{\mathrm{W}}{\mathrm{m.K}}$, respectively. We verified that our calculated heat flows were converged with respect to the spatial mesh.

The ON state calculations include a thermal contact resistance between the shuttle and the source-drain terminals; we fit this value to match the measured G_{on}, as discussed below. We fit the thermal contact resistance for our experimental data and obtained values ranging from $R_{\mathrm{contact}} = 0.9 * 10^{-3}$ to $2.2 * 10^{-3}\ \frac{\mathrm{K\,m^2}}{\mathrm{W}}$ for different measurements. These variations in R_{contact} could arise due to differences in the surface contact between the different trials, or partially due to uncertainties in the Q_{d} measurement associated with the thermocouple measurement variations. As one representative data point used in our discussion below, we obtained $R_{\mathrm{contact}} = 1.3 * 10^{-3}\ \frac{\mathrm{K\,m^2}}{\mathrm{W}}$ from the measurement in Fig. 2c obtained for $T_8 = 36.6^{\mathrm{o}}\mathrm{C}$, $T_1 = 17.1^{\mathrm{o}}\mathrm{C}$ and $T_{\mathrm{g}} = 45^{\mathrm{o}}\mathrm{C}$.

To interpret our measurements, we use a traditional model describing heat transfer between rough contacting surfaces in vacuum[1]. The predicted contact resistance for a unit area is

$$R_{\mathrm{contact}} = \left(1.25k\left(\frac{\theta}{\delta}\right)\left(\frac{P}{H}\right)^{0.95}\right)^{-1}, \qquad \text{Eq. (S1)}$$

where $H = 1225$ MPa is the microhardness, $\delta = 117$ nm is the surface roughness, $k = 400\ \frac{\mathrm{W}}{\mathrm{m.K}}$ is the thermal conductivity, $P = 5$ kPa is the contact pressure, and $\theta = 0.0084$ rad. is the asperity

slope. δ and θ were experimentally determined using optical profilometry on a polished copper surface that is similar to the copper surfaces used for our measurements. The contact pressure was estimated from FEM magnetostatics calculations, which provided an estimate for the magnetic force between the shuttle and the source drain terminals. These input values lead to an estimate of $R_{\text{contact}} = 3.7 * 10^{-3} \frac{\text{m}^2\text{K}}{\text{W}}$, which overestimates our measured value by a factor of ~3. This overprediction is likely due to our use of graphite foils as thermal interface materials (TIM), which would lower R_{contact} by providing additional conduction pathways between rough surfaces. In the ON state, this thermal contact resistance between the shuttle and the source-drain terminals is the dominant thermal resistance limiting the overall ON state performance of the transistor. The main strategies to minimize R_{contact} include increasing the contact pressure by increasing the magnetic forces, using TIMs with lower thermal resistance, or reducing the surface roughness of the contacting surfaces.

To find the dominant heat transfer modes in the OFF state in vacuum, we first study a high-T_s scenario using the Fig. 2c scenario with $T_8 = 63.7^\text{o}\text{C}$, $T_1 = 12.5^\text{o}\text{C}$ and $T_\text{g} = 10^\text{o}\text{C}$. For this case we find that the source-drain heat transfer is 57% through the polycarbonate support and 43% through parasitic radiation across the vacuum gap. In contrast, in a low-T_s trial with $T_8 = 23.4^\text{o}\text{C}$ and $T_1 = 14.7^\text{o}\text{C}$ and $T_\text{g} = 10^\text{o}\text{C}$, 84% of the heat is transferred via conduction through the support while only 16% is through radiation. These results show that the dominant parasitic heat transfer is conduction through the support, although radiation is not negligible at larger temperatures. For the transient measurements performed in air, the thermal bias is selected as to avoid natural convection currents. The thermal resistance due to conduction through the stagnant air layer between the source and drain is estimated as $R_{\text{air}} = 590 \frac{\text{K}}{\text{W}}$, which is smaller than the polycarbonate support thermal resistance $R_{\text{pc}} = 1800 \frac{\text{K}}{\text{W}}$, indicating that heat primarily is transferred via the air in measurements performed in ambient conditions.

Supplementary Note 2: Uncertainty analysis for reference bar apparatus

In the reference bar apparatus, eight steady-state temperatures along the reference bars were recorded for each data point shown in Fig. 2c. The uncertainty in Q_d was calculated from the error associated with fitting a slope to the temperature readings to extract the temperature gradient $m \equiv$

$\frac{dT}{dz}$. Following standard procedures, we find this slope m and the standard error of the slope σ_m in the upper and lower reference bars by minimizing the least-squared error as

$$\sigma_m = \frac{\sqrt{\frac{1}{N-2}\sum_{i=1}^{N}(T_i - (mz_i + b))^2}}{\sqrt{\sum_{i=1}^{N}(z_i - \bar{z})^2}}, \qquad \text{Eq. (S2)}$$

where i indexes the thermocouple measurement, N is the number of thermocouple measurements used to find the slope (here $N = 4$), T_i is the steady-state temperature measured at location z_i, $\bar{z} = \sum_{i=1}^{N} z_i/N$ is the average thermocouple location, and b is the extrapolated temperature at $z = 0$.

The values of m for the upper and lower reference bar differ due to parasitic losses via radiation to the environment. The ON state displays an average difference of 22% between the upper and lower reference bar slopes, while the OFF state average difference is 43%. As expected, the parasitic losses are more prevalent in the OFF state, leading to a larger variation in m. These measured values are in general agreement with the trends of our FEM calculations which displayed a 10% deviation in the ON state slopes for $T_8 = 50^{\circ}$C and $T_1 = 18^{\circ}$C and a 36% deviation in the OFF state slopes for $T_8 = 23^{\circ}$C and $T_1 = 15^{\circ}$C.

To calculate the thermal conductance, we perform a least-squares fit of G_{on} and G_{off} as the slopes of the ΔT_{sd} and Q_{d} plot shown in Fig. 2c; there is no offset in these linear fits, meaning that $Q_{\text{d}} = 0$ W when $\Delta T_{\text{sd}} = 0^{\circ}$C. The resulting values are $G_{\text{on}} \pm \sigma_{\text{on}} = 0.15 \pm 0.04\ \frac{\text{W}}{\text{K}}$ and $G_{\text{off}} \pm \sigma_{\text{off}} = 1.3 \pm 0.37\ \frac{\text{mW}}{\text{K}}$. We then calculate the switch ratio $G_{\text{on}}/G_{\text{off}} = 109 \pm 44$, where the uncertainty in the switch ratio is found using standard propagation of error as $\frac{G_{\text{on}}}{G_{\text{off}}}\sqrt{\left(\frac{\sigma_{\text{on}}}{G_{\text{on}}}\right)^2 + \left(\frac{\sigma_{\text{off}}}{G_{\text{off}}}\right)^2}$.

Supplementary Note 3: Transition temperature measurements

Supplementary Figure 1 shows our thermal measurements to quantify the transition temperatures $T_{\text{off-on}}$ and $T_{\text{on-off}}$. For a fixed OFF state gate-shuttle distance d (which is identical to the ON state shuttle-source/drain distance, as shown in the Supplementary Fig. 1 inset), we measured the steady-state temperatures at which the transistor fully switched from ON to OFF state and from OFF to ON state. The gate temperature T_{g} was slowly varied by controlling the power supplied to

the Peltier module placed on the gate, and we used visual observation of shuttle location to indicate switching.

Supplementary Figure 1 shows that the thermal deadband increases with gate distance. Focusing first on $T_{\mathrm{on-off}}$ (blue), lower gate temperatures are needed to switch the transistor OFF when the gate is far from the shuttle, because the magnetic forces between the shuttle Nd and gate Gd magnets decay with increasing d at fixed T_g. The larger Gd magnetic susceptibilities observed at lower T_{g} are therefore needed to actuate switching from ON-OFF, which reduces $T_{\mathrm{on-off}}$. In contrast, $T_{\mathrm{off-on}}$ (red) increases with increasing d because the Nd magnets on the source/drain are farther from the Nd magnets on the shuttle at large d. Because the Nd-Nd attractive force decreases with increasing d, larger T_{g} are needed to reduce the Gd magnetic susceptibility and achieve OFF-to-ON switching. The net result is that the deadband ($T_{\mathrm{off-on}} - T_{\mathrm{on-off}}$) increases from $6^{\mathrm{o}}\mathrm{C}$ at $d =$ 0.7 mm to $33^{\mathrm{o}}\mathrm{C}$ at $d = 3.8$ mm.

Supplementary Note 4: Differential thermal resistance discussion

The magnetic transistor displays negative differential thermal resistance R', which enables thermal amplification. To further explain the utility of the differential thermal resistance concept, and to emphasize the differences between the magnetic thermal transistor and traditional linear thermal systems, consider the three-terminal arrangement shown in Supplementary Figure 5c. This system consists of three thermal resistors with resistance R_1, R_2, and R_3. Each resistor has one of its terminals connected to a thermal reservoir at temperatures T_1, T_2, and T_3, and the other terminal of the resistors is at a shared and unknown temperature T_0. The resistors are assumed to be thermally linear, meaning that $Q_1 = (T_1 - T_0)/R_1$, $Q_2 = (T_2 - T_0)/R_2$, and $Q_3 = (T_0 - T_3)/R_3$, where Q_1 and Q_2 are the heat flows entering the network at T_1 and T_2, respectively, while Q_3 is the heat flow leaving the network at T_3.

We solve for T_0 by combining these constitutive relationships with the energy balance requirement $Q_1 + Q_2 = Q_3$ and find

$$T_0 = \frac{\left(\frac{T_1}{R_1} + \frac{T_2}{R_2} + \frac{T_3}{R_3}\right)}{R_1^{-1} + R_2^{-1} + R_3^{-1}}. \quad \text{Eq. (S3)}$$

Inserting this junction temperature into the relation $Q_1 = (T_1 - T_0)/R_1$ and simplifying the resulting expression, the heat flow into terminal 1 is

$$Q_1 = \frac{\left(\frac{T_1 - T_2}{R_2} + \frac{T_1 - T_3}{R_3}\right)}{1 + \frac{R_1}{R_2} + \frac{R_1}{R_3}}. \qquad \text{Eq. (S4)}$$

Eq. (S4) shows that increasing T_2 or T_3 at fixed T_1 decreases Q_1, because the driving temperature difference $(T_1 - T_0)$ becomes less positive as T_2 or T_3 increases while R_1 is independent of T. In this sense, T_2 does have an influence on Q_1, as has been shown in a prior demonstration of a three-terminal thermal device[2]; however, this is not sufficient to achieve thermal transistor action. Introducing the differential thermal resistance of terminal 1 as $R_1' = -\left(\frac{dQ_1}{dT_2}\right)^{-1}\Big|_{T_1,T_3}$ and using Eq. (S4), we see that

$$R'_1 = \left(R_1 + R_2 + \frac{R_1 R_2}{R_3}\right). \qquad \text{Eq. (S5)}$$

This differential resistance is positive valued because the thermal resistances R_1, R_2, and R_3 must all be positive to satisfy the second law of thermodynamics. This calculation emphasizes that the negative differential resistance of the magnetic transistor is qualitatively distinct from the behavior of a simple linear system, as expected for a thermal transistor. Similarly, the differential thermal resistance of terminal 3 is defined as $R_3' = \left(\frac{dQ_3}{dT_2}\right)^{-1}\Big|_{T_1,T_3}$ and found from the above analysis as

$$R'_3 = \left(R_3 + R_2 + \frac{R_2 R_3}{R_1}\right). \qquad \text{Eq. (S6)}$$

The previous derivation by Li, Wang, and Castelli[3], further assumed that $R_2 \ll R_1$ and $R_2 \ll R_3$, leading to the simpler results that $R'_1 = R_1$ and $R'_3 = R_3$.

We now confirm that this system of thermal resistors with positive differential thermal resistance does not display thermal amplification for any choice of thermal resistances, temperatures, or heat flows. Returning to Eq. (S3), we can rewrite the expression for T_0 in terms of Q_2 as

$$T_0 = \frac{\left(\frac{T_1}{R_1} + Q_2 + \frac{T_3}{R_3}\right)}{R_1^{-1} + R_3^{-1}}. \qquad \text{Eq. (S7)}$$

Similarly, we can rewrite the Eq. (S4) expression for Q_1 in terms of Q_2 as

$$Q_1 = \frac{\left(Q_2 + \frac{T_1 - T_3}{R_3}\right)}{1 + \left(\frac{R_1}{R_3}\right)}. \qquad \text{Eq. (S8)}$$

The amplification parameter $\beta = \left.\frac{dQ_1}{dQ_2}\right|_{T_1,T_3}$ is therefore

$$\beta = \frac{R_1}{R_1 + R_3}. \qquad \text{Eq. (S9)}$$

β is always bounded to be smaller than unity, confirming that the system cannot amplify heat flows. Under the small-R_2 assumption, the amplification is simply $R'_1/(R_1' + R_3')$, in agreement with the prior derivation[3]. This small-R_2 limit makes it clear that achieving amplification requires at least one of the differential thermal resistances R_1' or R_3' to be negative-valued, such that the denominator has a smaller magnitude than the numerator. Lastly, as an aside we note that similar small-signal amplification analysis and conclusions can also be obtained graphically using load-line methods, which have also been applied to the study of nonlinear thermal elements[4].

Supplementary Note 5: NOT Logic Gate Demonstration.

Supplementary Figure 8 shows that the thermal transistor can also be used to create a NOT thermal logic gate illustrated in Supplementary Fig. 8b. The NOT gate schematic of Supplementary Fig. 8a shows an input temperature T_1, which corresponds to the temperature of the gate, and an output temperature T_{out}, which corresponds to the transistor source temperature T_{s}. The thermal transistor is placed in series with a thermal resistance R_{s} between T_{max} and T_{min}. When T_1 is LOW, the transistor is OFF and the output temperature is driven HIGH towards T_{max}. When T_1 is HIGH, the transistor is ON and the output temperature is driven LOW towards T_{min}.

Supplementary Fig. 8c shows our measurements in air (black) and vacuum (red) of the dimensionless output temperature $\frac{T_{\mathrm{out}}-T_{\mathrm{min}}}{T_{\mathrm{max}}-T_{\mathrm{min}}}$ (top panel) for two different input temperatures T_1 (bottom panel). T_1 can have a Boolean value of 1 (HIGH) if $T_{\mathrm{g}} < T_{\mathrm{on-off}}$, and a Boolean value of

0 if $T_g > T_{off-on}$. As expected from a NOT gate and as shown by our experimental values, for HIGH T_1 the output will be LOW, and for LOW T_1 the output will be HIGH. In the experiment T_{min} was kept around 20^oC for both trials, whereas T_{max} was around 30^oC for the air trial and 55^oC for the vacuum trial. Our measurements indicate that the Boolean HIGH value is 0.91 in vacuum and 0.85 in air, whereas the Boolean LOW value is 0.21 in vacuum and 0.24 in air. These results show that the NOT gate performs slightly better in vacuum than air, due to the elimination of the parasitic conduction through the air layer in the OFF state.

Supplementary Fig. 8d and Supplementary Fig. 8e show the theoretical predictions for dimensionless temperatures as a function of switch ratio γ for four values of the dimensionless series resistance $\omega = \sqrt{G_{ON}G_{OFF}}R_s$. In the HIGH state, it is straightforward to show that $\frac{T_{out}-T_{min}}{T_{max}-T_{min}} = \frac{\sqrt{\gamma}}{(\omega+\sqrt{\gamma})}$, while in the LOW state, $\frac{T_{out}-T_{min}}{T_{max}-T_{min}} = \frac{1}{(\omega\sqrt{\gamma}+1)}$. Increasing γ is always desirable to increase the difference between the HIGH and LOW NOT values. At fixed γ, the difference between HIGH and LOW NOT gate values is optimized when the dimensionless series resistance $\omega = 1$. At this optimal series resistance, the difference in the HIGH-LOW state dimensionless temperatures is $\frac{\sqrt{\gamma}-1}{\sqrt{\gamma}+1}$, which is zero at $\gamma = 0$ and approaches the upper limit of unity at $\gamma \gg 1$. Using the above equations to extract γ and ω from the measured dimensionless temperature values in air and vacuum, we find that $\omega_{air} = 0.74$, $\gamma_{air} = 18$, $\omega_{vac} = 0.62$, and $\gamma_{vac} = 36$. These experimental values (red and black markers for vacuum and air, respectively) are shown in Supplementary Fig. 8d,e. This comparison shows that performance of the NOT gate is close to the $\omega = 1$ optimal scenario (green line). In our experimental setup, we used a rubber pad as a series resistor. In air, we used a thickness corresponding to $R_s = 12\frac{K}{W}$, while in vacuum we used $R_s = 24\frac{K}{W}$. Combining these R_s values with the known γ and ω shows that in these measurements, $G_{ON,air} = 0.26\frac{W}{K}$, $G_{OFF,air} = 0.015\frac{W}{K}$, $G_{ON,vac} = 0.16\frac{W}{K}$, and $G_{OFF,vac} = 0.0044\frac{W}{K}$. G_{ON} is higher in air than in vacuum because the microscopic gaps between the mating shuttle and source-drain terminals are filled with air pockets that increase the conductance. G_{OFF} is also larger in air than in vacuum, since there is additional conduction through the air layer in the OFF state.

Supplementary Note 6: Discussion of transistor nomenclature

Our discussion follows the standard definition of a thermal transistor as a three-terminal thermal element that switches and amplifies heat flows between two terminals via the temperature or heat flow at a third terminal[3,5]. The fundamental nature of the switching and amplification of our device is more analogous to the gate voltage-driven electrical Field Effect Transistor (FET) rather than the base-current driven electrical Bipolar Junction Transistor (BJT). We compare several aspects of these devices in Supplementary Table 3.

In a FET, the gate voltage controls the source-drain conductance and the steady-state gate current is only due to leakage. The FET would still operate well even if the gate current were reduced dramatically at fixed drain voltage (e.g. using better dielectrics to prevent current leakage) because the field effect is a voltage-driven phenomena, with the gate current as an unwanted byproduct to be minimized. Similarly, in our thermal transistor, the temperature of the gate terminal controls the source-drain conductance and the gate heat flow is only due to radiation between the gate terminal and the shuttle. Our thermal transistor would still operate well if the gate radiation heat flow were reduced dramatically at fixed gate temperature (e.g. by using IR reflective coatings to reduce the emissivity) because the actuation is due to a temperature-driven magnetic phase transition.

In a BJT, in contrast, the base current controls the emitter current via the bipolar effect, in which the position- and current-dependent populations of majority and minority carriers in the vicinity of *npn* or *pnp* junctions leads to amplification. BJTs also display electrical switching; for example, the collector-emitter conductance is OFF if the base voltage is the same as the emitter voltage and ON at large base currents. Crucially, if the base current were eliminated (e.g. if a perfect dielectric prevented carriers from being injected into the base), the device could not function as an electrical switch for any setting of the base voltage; it is in this sense that the BJT is a current-controlled rather than a voltage-controlled device.

We note that previously proposed thermal radiation transistors that leverage phase-change materials (e.g. VO_2) are also temperature-gated[5–11], because the nonlinearities arise due to the gate temperature-dependent optical properties. These phase-change transistors could still amplify and switch source-drain heat flows even if the gate heat flow was reduced in both ON and OFF states. The fact that these previous simulation papers on far-field radiative thermal transistors using VO_2 have also calculated heat flow amplification in macroscale systems emphasizes that thermal

amplification could be observed in several macroscopic thermal devices, including the thermomagnetic devices investigated in this work.

It is interesting to compare the thermal transistors against familiar thermodynamic heat pump/refrigeration cycles. In a thermal transistor, the heat flow amplification refers to the ratio of the change in the drain heat flow to the change in the gate heat flow. Because thermal energy is always transferred from hot to cold reservoirs in the thermal transistor, there is no thermodynamic limitation on the heat flow amplification ratio. In contrast, the net flow of thermal energy in a heat pump/refrigeration cycle occurs from the cold reservoir to the hot reservoir, and the coefficient of performance (ratio of the desired heat flow to the input electrical power) is thermodynamically bounded by the Carnot limit.

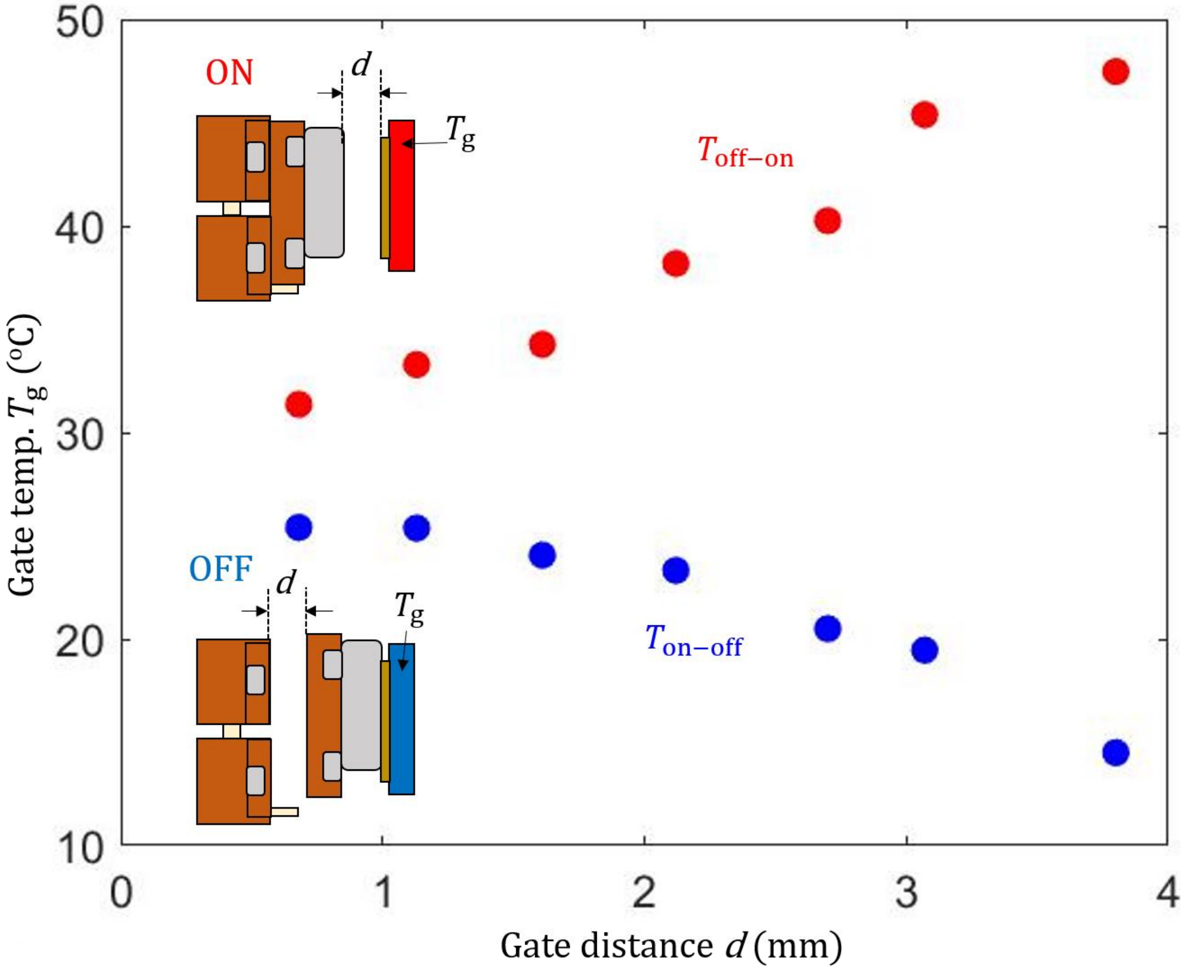

Supplementary Figure 1: Our measurements show that the switching temperatures and deadband can be controlled by changing the distance d between the gate terminal and the shuttle in the OFF state (see inset schematic). $T_{\mathrm{off-on}}$ (red) increases with d while $T_{\mathrm{on-off}}$ (blue) decreases with d, leading to larger thermal hysteresis at larger d.

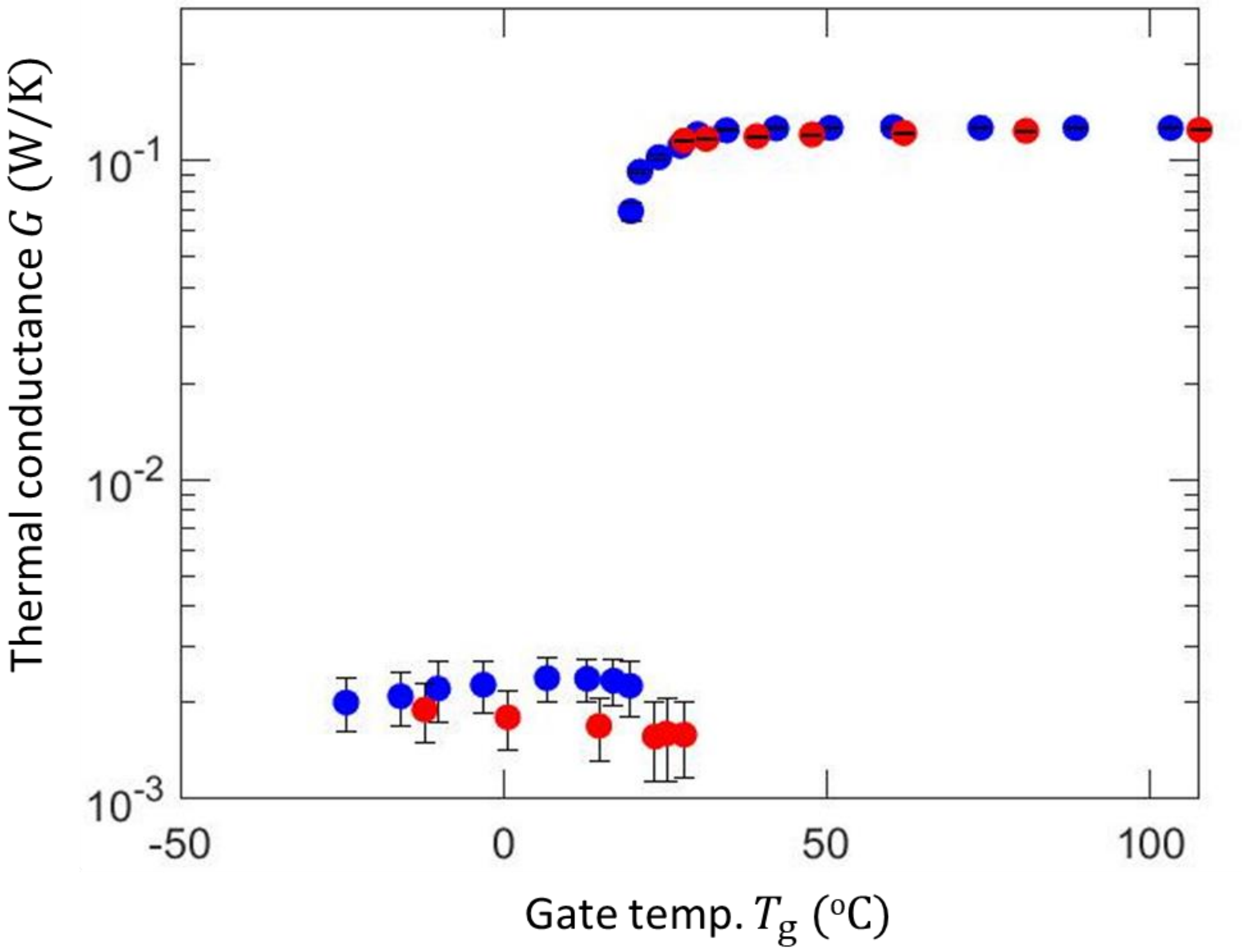

Supplementary Figure 2. Thermal conductance measurements extracted from Fig. 2d upon heating (red) and cooling (blue) as T_g varies from -25^oC to 110^oC. Error bars represent standard deviation of G; the uncertainties for Q_d and ΔT_{sd} are calculated as outlined in Supplementary Note 2 and propagated into the uncertainty in G.

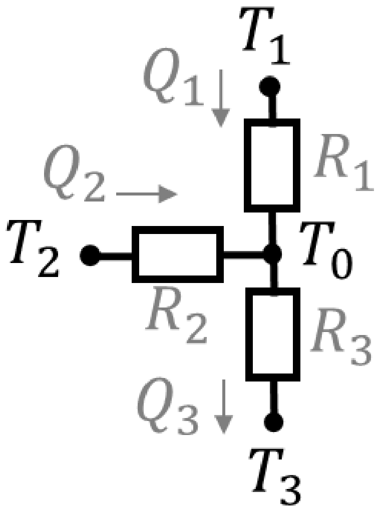

Supplementary Figure 3. Thermal circuit of three-terminal thermal resistor system. Unlike the magnetic thermal transistor, this linear system displays the traditional positive differential thermal resistance $R' > 0$ and cannot be used for thermal amplification.

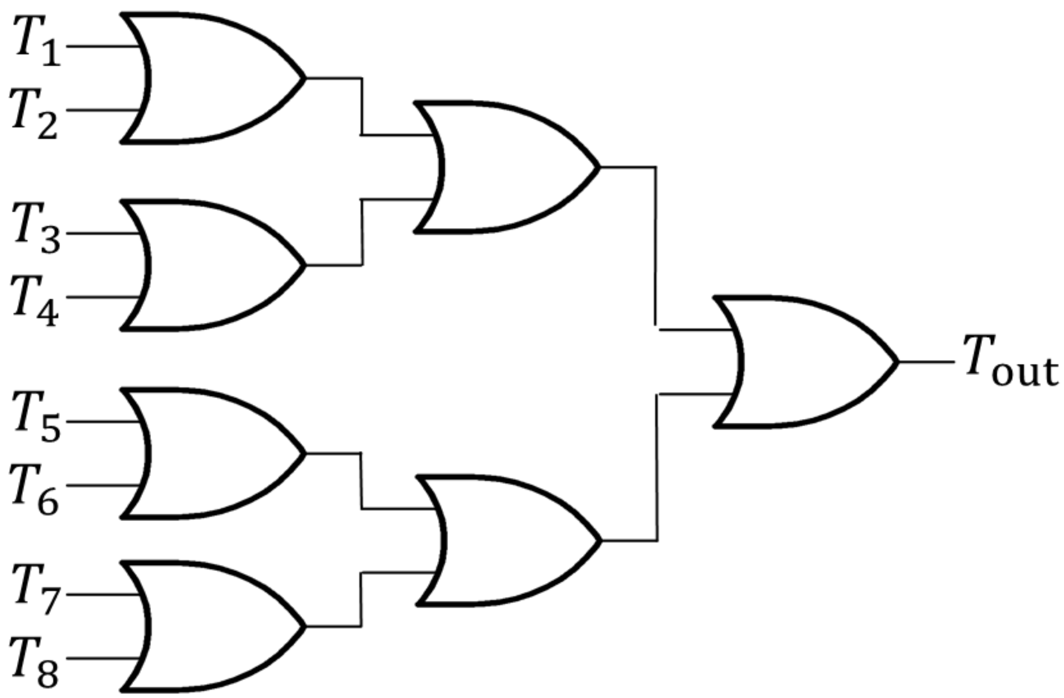

Supplementary Figure 4. Cascaded thermal OR gate circuit in which the output temperature T_{out} is Boolean HIGH if any of the input temperatures $T_1 - T_8$ are HIGH, and LOW otherwise. The circuit could be used to detect overheating at any of the input elements with a single thermal readout T_{out}.

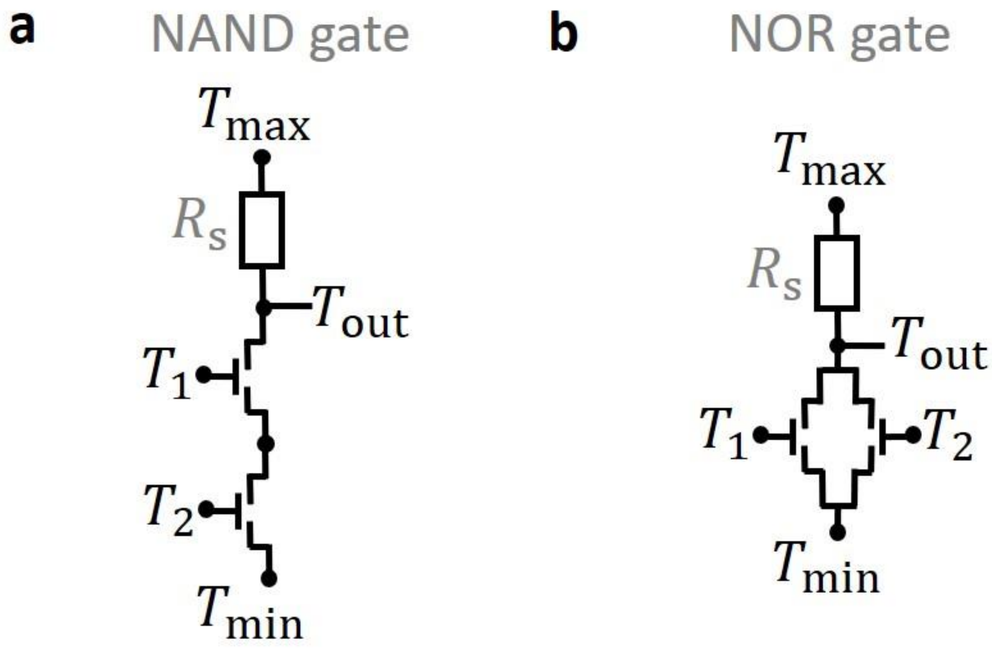

Supplementary Figure 5. Transistor thermal circuit representations for two additional thermal logic gates. (a) Thermal NAND gate. The output is LOW if T_1 and T_2 are both HIGH (above the switching temperature), and the output is HIGH if one or both of T_1 and T_2 are LOW (below the switching temperature). (b) Thermal NOR gate. The output is LOW if either of T_1 and T_2 are HIGH, and the output is HIGH if T_1 and T_2 are both LOW.

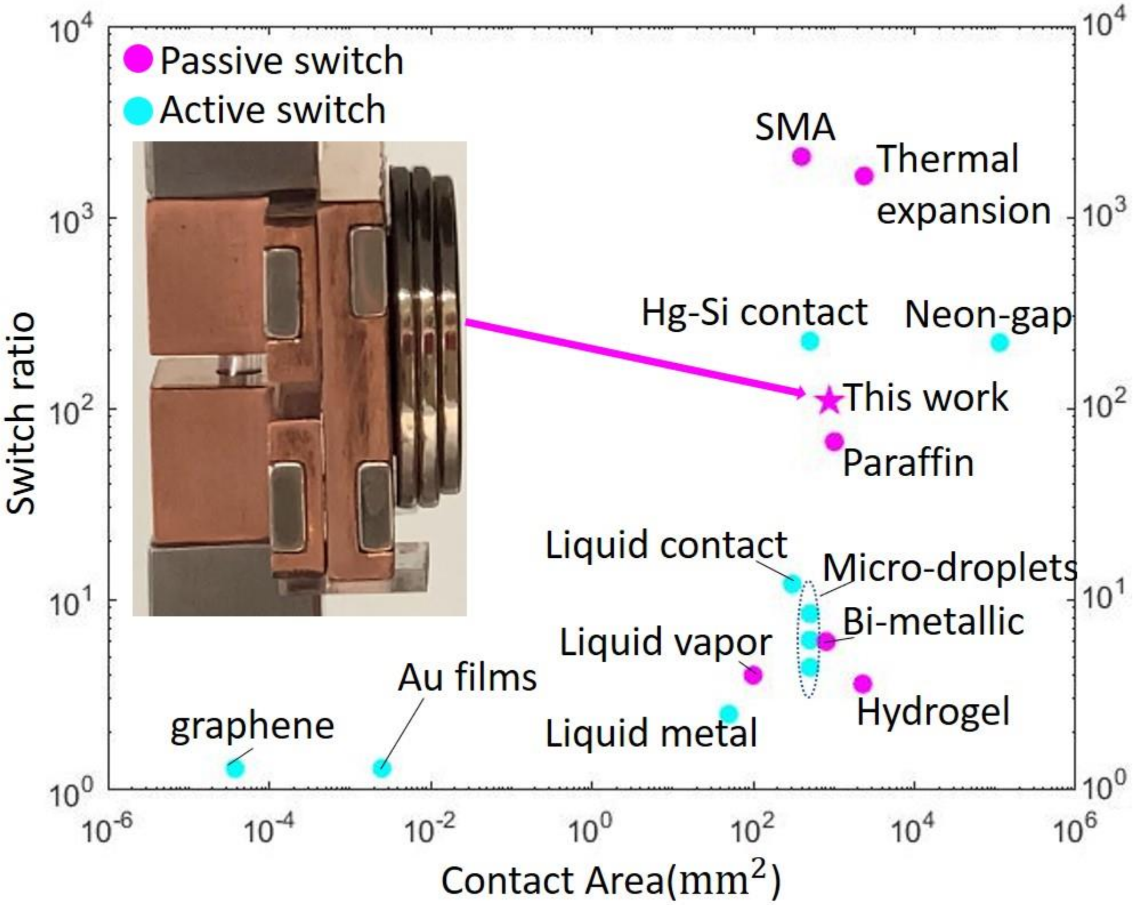

Supplementary Figure 6. Thermal switch ratio comparison between two-terminal contact-based passive (magenta circles) and active (cyan circles) thermal switches and the three-terminal magnetic thermal transistor (magenta star). Switches with larger contact areas often have larger switch ratios because parasitic conduction through the supports is a large contribution to G_{off} in small-contact thermal switches. All selected switches are contact-based devices in which the contact material is either a solid or a fluid[12-24].

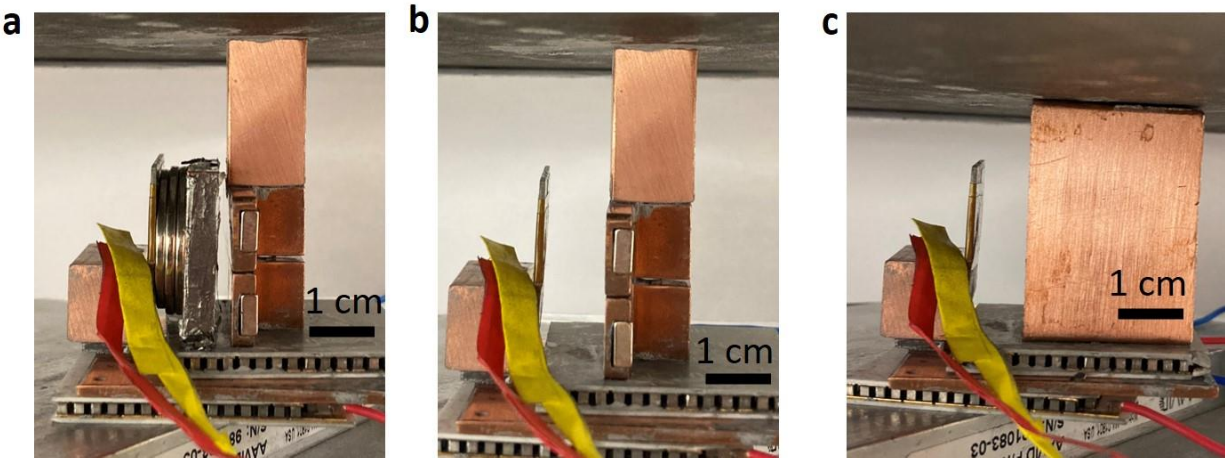

Supplementary Figure 7. Passive heat routing experiment setup used for measurements shown in Fig. 5e. (a) Optical image of setup for the transistor in series with the TEG generator, implementing the thermal circuit shown in Fig. 5c. (b) Optical image of high-R_{sd} implementation of thermal circuit in Fig. 5d, in which the shuttle is removed from the transistor. (c) Optical image of low-R_{sd} implementation of thermal circuit in Fig. 5d in which the transistor is replaced by a solid copper block.

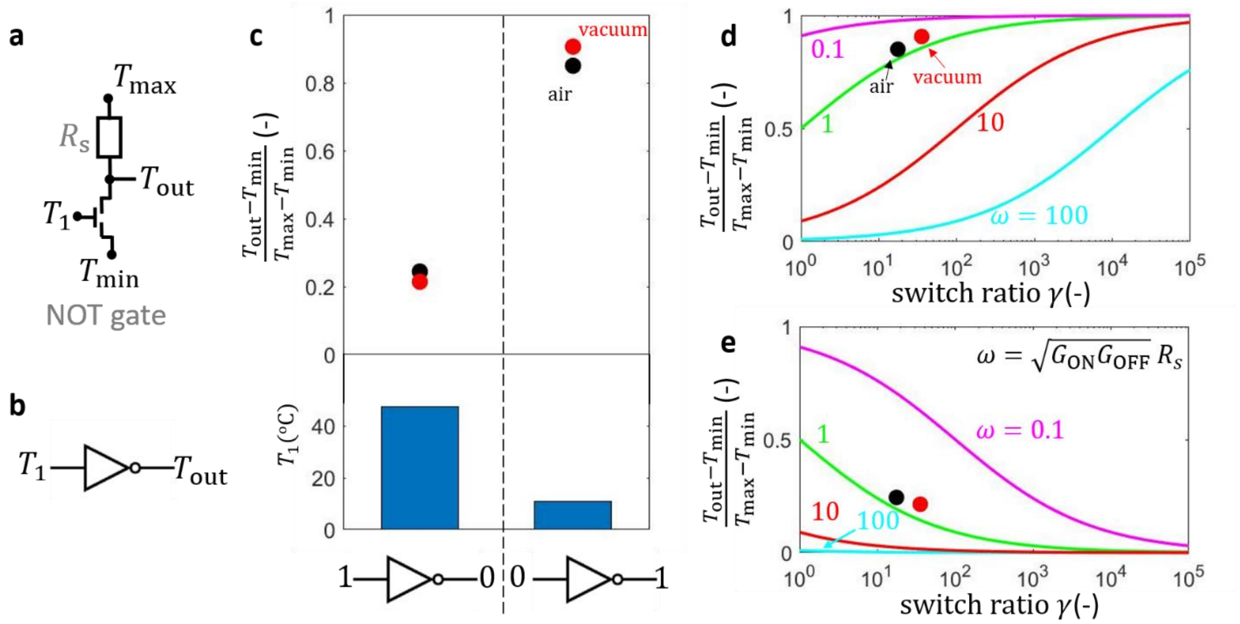

Supplementary Figure 8: Thermal NOT gate. (a) Thermal circuit schematic and (b) logic gate representation of thermal NOT gate using one transistor between a maximum supply temperature $T_{\max}$ and minimum supply temperature $T_{\min}$. The input temperature T_1 is connected to the gate terminal, and the output temperature T_{out} is separated from $T_{\max}$ by a series thermal resistor R_s. (c) Measured NOT gate truth table. The output normalized temperature is a Boolean 1 (HIGH) if $T_1 < T_{\text{on-off}}$, while the output temperature is a Boolean 0 (LOW) for $T_1 > T_{\text{off-on}}$. The gate performs slightly better in vacuum (red) than in air (black) due to a larger switch ratio in vacuum. (d) Thermal modeling shows that the NOT gate performance depends only on the switch ratio γ and the dimensionless series resistance ω. For low T_1 (Boolean 1), the dimensionless output temperature increases with increasing γ for four ω ranging from 0.1 to 100 (lines). Our experimental measurements from (c) are similar to the optimal $\omega = 1$ scenario (green line) in both air and vacuum. (e) For high T_1 (Boolean 0), the output temperature decreases with increasing γ for the same ω as shown in (d). The HIGH-LOW temperature difference at the optimal series resistance $\omega = 1$ is $\frac{\sqrt{\gamma}-1}{\sqrt{\gamma}+1}$, showing that large γ are essential for NOT gate performance.

Supplementary Table 1: ON-state reference bar measurements for seven thermal bias conditions. $T_1 - T_8$ are steady-state thermocouple measurements, while $T_\mathrm{s}, T_\mathrm{d}, \Delta T_\mathrm{sd}$ and Q_d are extracted values.

Quantity	Unit	#1	#2	#3	#4	#5	#6	#7
T_8	^{o}C	24.44	29.12	30.53	36.62	39.24	50.10	33.39
T_7	^{o}C	23.31	27.59	28.55	34.26	36.33	46.03	31.88
T_6	^{o}C	22.11	26.14	26.64	31.91	33.54	41.98	30.12
T_5	^{o}C	20.96	24.57	24.82	29.63	30.94	38.31	28.71
T_4	^{o}C	17.76	20.05	19.64	22.45	22.95	26.95	21.41
T_3	^{o}C	17.19	19.18	18.72	21.02	21.32	24.35	20.08
T_2	^{o}C	16.18	17.77	17.35	19.03	19.16	21.41	18.84
T_1	^{o}C	14.98	16.36	15.74	17.10	16.77	18.14	16.97
T_s	^{o}C	19.79	23.06	22.91	27.30	28.17	34.39	27.15
T_d	^{o}C	18.69	21.27	20.94	24.23	25.01	29.89	22.89
ΔT_sd	^{o}C	1.10	1.78	1.97	3.07	3.16	4.50	4.26
Q_d	W	0.24	0.32	0.34	0.46	0.53	0.76	0.37

Supplementary Table 2: OFF-state reference bar measurements for eight thermal bias conditions. $T_1 - T_8$ are steady-state thermocouple measurements, while $T_\mathrm{s}, T_\mathrm{d}, \Delta T_\mathrm{sd}$ and Q_d are extracted values.

Quantity	Unit	#1	#2	#3	#4	#5	#6	#7	#8
T_8	°C	23.43	23.60	26.29	31.15	34.38	41.20	44.86	63.72
T_7	°C	23.42	23.43	26.25	31.08	34.23	40.96	44.41	63.10
T_6	°C	23.40	23.24	26.13	30.81	33.97	40.40	44.05	63.04
T_5	°C	23.27	23.15	25.96	30.77	33.64	40.36	43.49	62.26
T_4	°C	14.96	13.22	14.79	14.39	14.75	14.45	14.81	13.32
T_3	°C	14.91	13.18	14.66	14.33	14.50	14.25	14.74	13.16
T_2	°C	14.86	13.16	14.63	14.29	14.37	14.17	14.55	13.02
T_1	°C	14.66	12.86	14.40	13.99	14.34	13.85	14.47	12.51
T_s	°C	23.21	23.00	25.86	30.65	33.40	40.08	43.03	61.77
T_d	°C	15.06	13.34	14.92	14.53	14.89	14.65	14.92	13.59
ΔT_sd	°C	8.15	9.66	10.94	16.12	18.51	25.42	28.12	48.18
Q_d	W	0.019	0.022	0.026	0.027	0.028	0.044	0.022	0.060

Supplementary Table 3: Comparison between electrical field effect transistors (FETs), this work's thermal transistor demonstration, and electrical bipolar junction transistors (BJT).

**Device**	**FET**	**This work**	**BJT**
**Circuit diagram representation**	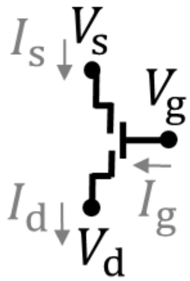	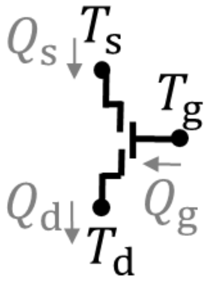	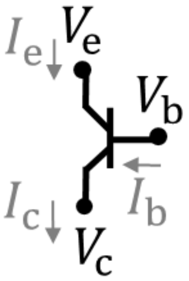
**Physical mechanism of transistor action**	Gate voltage controls source-drain electrical conductance via field effect	Gate temperature controls source-drain thermal conductance via shuttle position	Base current controls emitter current via bipolar effects near *npn* or *pnp* junctions
**Gate/base current origin**	Gate current is due to leakage (seek to minimize)	Gate heat flow is due to radiation (seek to minimize)	Base current is intentionally injected to achieve bipolar effect.

Supplementary References

1. Yovanovich, M. M. Four decades of research on thermal contact, gap, and joint resistance in microelectronics. *IEEE Trans. Components Packag. Technol.* **28**, 182–206 (2005).

2. Gou, X., Ping, H., Ou, Q., Xiao, H. & Qing, S. A novel thermoelectric generation system with thermal switch. *Appl. Energy* **160**, 843–852 (2015).

3. Li, B., Wang, L. & Casati, G. Negative differential thermal resistance and thermal transistor. *Appl. Phys. Lett.* **88**, 143501 (2006).

4. Wehmeyer, G., Yabuki, T., Monachon, C., Wu, J. & Dames, C. Thermal diodes, regulators, and switches: Physical mechanisms and potential applications. *Appl. Phys. Rev.* **4**, 041304 (2017).

5. Joulain, K., Ezzahri, Y., Drevillon, J. & Ben-abdallah, P. Modulation and amplification of radiative far field heat transfer : Towards a simple radiative thermal transistor. *Appl. Phys. Lett.* **106**, 133505 (2015).

6. Ben-Abdallah, P. & Biehs, S. A. Near-field thermal transistor. *Phys. Rev. Lett.* **112**, 044301 (2014).

7. Chen, F., Liu, X., Tian, Y., Wang, D. & Zheng, Y. Non-contact thermal transistor effects modulated by nanoscale mechanical deformation. *J. Quant. Spectrosc. Radiat. Transf.* **259**, 107414 (2021).

8. Latella, I., Marconot, O., Sylvestre, J., Fréchette, L. G. & Ben-abdallah, P. Dynamical Response of a Radiative Thermal Transistor Based on Suspended Insulator-Metal-Transition Membranes. *Phys. Rev. Appl.* **11**, 024004 (2019).

9. Prod, H., Ordonez-miranda, J., Ezzahri, Y., Drévillon, J. & Joulain, K. VO2-based radiative thermal transistor with a semi-transparent base. *J. Quant. Spectrosc. Radiat. Transf.* **210**, 52–61 (2018).

10. Hugo, P., Ordonez-Miranda, J. & Ezzahri, Y. Optimized thermal amplification in a radiative transistor. *J. Appl. Phys.* **119**, 194502 (2016).

11. Ordonez-Miranda, J., Ezzahri, Y., Drevillon, J. & Joulain, K. Dynamical heat transport

amplification in a far-field thermal transistor of VO2 excited with a laser of modulated intensity. *J. Appl. Phys.* **119**, 203105 (2016).

12. Cho, J., Richards, C., Bahr, D., Jiao, J. & Richards, R. Evaluation of contacts for a MEMS thermal switch. *J. Micromechanics Microengineering* **18**, 105012 (2008).

13. Hao, M., Li, J., Park, S., Moura, S. & Dames, C. Efficient thermal management of Li-ion batteries with a passive interfacial thermal regulator based on a shape memory alloy. *Nat. Energy* **3**, 899–906 (2018).

14. Bugby, D. C. & Rivera, J. G. 2020 International Conference on Environmental Systems. *Ices* **145**, 1–10 (2020).

15. Sunada, E. *et al.* Design and flight qualification of a paraffin-actuated heat switch for Mars surface applications. *SAE Int.* **111**, 202–207 (2002).

16. Catarino, I., Bonfait, G. & Duband, L. Neon gas-gap heat switch. *Cryogenics (Guildf).* **48**, 17–25 (2008).

17. Yang, T. *et al.* An Integrated Liquid Metal Thermal Switch for Active Thermal Management of Electronics. *IEEE Trans. Components Packag. Technol.* **9**, 2341–2351 (2019).

18. Miao, R., Kishore, R., Kaur, S. & Prasher, R. A non-volatile thermal switch for building energy savings. *Cell Reports Phys. Sci.* **3**, 100960.

19. Mclanahan, A. R., Richards, C. D. & Richards, R. F. A dielectric liquid contact thermal switch with electrowetting actuation. *J. Micromechanics Microengineering* **21**, 104009 (2011).

20. Liu, T. *et al.* Tunable, passive thermal regulation through liquid to vapor phase change. *Appl. Phys. Lett.* **115**, 254102 (2019).

21. Milanez, F. H. & Mantelli, M. B. H. Theoretical and experimental studies of a bi-metallic heat switch for space applications. *Int. J. Heat Mass Transf.* **46**, 4573–4586 (2003).

22. Feng, H. *et al.* Thermally-Responsive Hydrogels Poly (N - Isopropylacrylamide) as the Thermal Switch. *J. Phys. Chem. C* **123**, 31003 (2019).

23. Keum, H., Seong, M., Sinha, S. & Kim, S. Electrostatically driven collapsible Au thin films assembled using transfer printing for thermal switching. *Appl. Phys. Lett.* **100**, 211904 (2012).

24. Chen, M. E. *et al.* Graphene-based electromechanical thermal switches. *2D Mater.* **8**, 035055 (2021).
