# Supplementary material for: A three-terminal magnetic thermal transistor
Source: Nat Commun. 2023 Jan 24;14:393. doi: 10.1038/s41467-023-36056-4 (PMC9873738; doi:10.1038/s41467-023-36056-4)
Supplement: Supplementary file 2 — Description of Additional Supplementary Files [file 41467_2023_36056_MOESM2_ESM.pdf]

Description of Additional Supplementary Files

Supplementary Movie 1. Video of gate-temperature driven thermal transistor switching
